# Premature senescence and cardiovascular disease following cancer treatments: mechanistic insights

**DOI:** 10.3389/fcvm.2023.1212174

**Published:** 2023-09-14

**Authors:** Ashita Jain, Diego Casanova, Alejandra Valdivia Padilla, Angelica Paniagua Bojorges, Sivareddy Kotla, Kyung Ae Ko, Venkata S. K. Samanthapudi, Khanh Chau, Minh T. H. Nguyen, Jake Wen, Selina L. Hernandez Gonzalez, Shaefali P. Rodgers, Elizabeth A. Olmsted-Davis, Dale J. Hamilton, Cielito Reyes-Gibby, Sai-Ching J. Yeung, John P. Cooke, Joerg Herrmann, Eduardo N. Chini, Xiaolei Xu, Syed Wamique Yusuf, Momoko Yoshimoto, Philip L. Lorenzi, Brain Hobbs, Sunil Krishnan, Efstratios Koutroumpakis, Nicolas L. Palaskas, Guangyu Wang, Anita Deswal, Steven H. Lin, Jun-ichi Abe, Nhat-Tu Le

**Affiliations:** ^1^ Department of Cardiology, The University of Texas MD Anderson Cancer Center, Houston, TX, United States; ^2^ Department of Cardiovascular Sciences, Houston Methodist Research Institute, Houston, TX, United States; ^3^ Department of Medicine, Center for Bioenergetics, Houston Methodist Research Institute, Houston, TX, United States; ^4^ Department of Emergency Medicine, The University of Texas MD Anderson Cancer Center, Houston, TX, United States; ^5^ Cardio Oncology Clinic, Division of Preventive Cardiology, Department of Cardiovascular Medicine, Mayo Clinic, Rochester, MN, United States; ^6^ Department of Anesthesiology and Perioperative Medicine, Mayo Clinic, Jacksonville, FL, United States; ^7^ Department of Cardiovascular Medicine, Mayo Clinic, Rochester, MN, United States; ^8^ Center for Stem Cell & Regenerative Medicine, The University of Texas Health Science Center at Houston, Houston, TX, United States; ^9^ Department of Bioinformatics and Computational Biology, Division of VP Research, The University of Texas MD Anderson Cancer Center, Houston, TX, United States; ^10^ Department of Population Health, The University of Texas at Austin, Austin, TX, United States; ^11^ Department of Neurosurgery, The University of Texas Health Science Center at Houston, Houston, TX, United States; ^12^ Department of Radiation Oncology, The University of Texas MD Anderson Cancer Center, Houston, TX, United States

**Keywords:** premature senescence, cardio-oncology, DNA damage, telomere dysfunction, mitochondrial dysfunction, fission and fusion, autophagy, NAD^+^

## Abstract

Cardiovascular disease (CVD) is a leading cause of morbidity and mortality, especially among the aging population. The “response-to-injury” model proposed by Dr. Russell Ross in 1999 emphasizes inflammation as a critical factor in atherosclerosis development, with atherosclerotic plaques forming due to endothelial cell (EC) injury, followed by myeloid cell adhesion and invasion into the blood vessel walls. Recent evidence indicates that cancer and its treatments can lead to long-term complications, including CVD. Cellular senescence, a hallmark of aging, is implicated in CVD pathogenesis, particularly in cancer survivors. However, the precise mechanisms linking premature senescence to CVD in cancer survivors remain poorly understood. This article aims to provide mechanistic insights into this association and propose future directions to better comprehend this complex interplay.

## 1. Introduction

The advent of advanced cancer treatments has revolutionized cancer management, offering new possibilities for individuals with various types of cancer. Screening, early detection, and treatment modalities such as targeted therapies, immunotherapy, radiation therapy (RT), and chemotherapy have significantly improved patient outcomes and survival rates. However, understanding the potential long-term effects and complications associated with these treatments is crucial. We need to explore the underlying mechanisms involved in cellular communication, treatment response, and systemic effects to develop targeted interventions and personalized approaches, optimizing the long-term health of cancer survivors (1–4).

### 1.1. CVD associated with cancer treatments

#### 1.1.1. Drawbacks of advanced cancer treatments

Cancer management has witnessed remarkable advancements, with targeted therapies playing a crucial role in various types of cancer treatment. For example, sorafenib has been instrumental in hepatocellular carcinoma treatment (5), while drugs like sunitinib, sorafenib, temsirolimus, and bevacizumab have shown effectiveness in renal cell carcinoma (6). Bevacizumab, cetuximab, and panitumumab have shown benefit in colon cancer (7–9), and cetuximab has shown promise in head and neck cancers (10). In pancreatic cancer, erlotinib has yielded positive outcomes (11, 12). Undoubtedly, these targeted therapies have revolutionized treatment for their respective cancer types (13).

Novel targeted therapies has shown promise in overcoming treatment resistance. For example, lapatinib has proven effective in breast cancer cases resistant to other targeted therapies (14). Similarly, ponatinib and dasatinib have exhibited positive outcomes in chronic myeloid leukemia patients, addressing resistance concerns (15, 16). Monoclonal antibodies like rituximab, has been successful either as standalone treatments or in combination with chemotherapy, particularly in managing indolent lymphoma (17, 18). Furthermore, the incorporation of imatinib in the adjuvant setting for resected gastrointestinal stromal tumors has improved outcomes for these cases (19–25). Chemotherapeutic agents like oxaliplatin has enhanced survival rates for specific cancer types, such as colon cancer (26), while temozolomide has shown promise in treating malignant glioma (27, 28). Adjuvant chemotherapy has also yielded positive results in non-small cell lung cancer cases (29, 30).

Advancements in cancer screening and early detection, along with treatments such as targeted therapies, immunotherapy, RT, and chemotherapy (13, 29) have significantly improved cancer survival rates. However, it is important to recognize that cancer treatments can lead to long-term and latent effects (31, 32). Long-term effects emerge during treatment and persist over time, while latent effects may only appear years after treatment completion. A comprehensive review by Gegechkori et al. (2017) (33) summarizes common long-term and latent treatment effects associated with prevalent cancers. As the number of cancer survivors continues to increase, understanding and managing these treatment-related effects becomes more critical. In the United States alone, there are currently over 15 million cancer survivors, projected to exceed 20 million by 2026 (34). While many survivors may face other comorbidities overtime, it is crucial to acknowledge the lasting consequences of the treatments they underwent.

#### 1.1.2. CVD associated with cancer treatments

Certain cancer treatments, such as RT (35–39) and chemotherapy (40, 41), are linked to an increased risk of cardiovascular complications (42, 43), which may manifest years or decades after treatment completion (44). These complications encompass various cardiovascular conditions including hypertension, arrhythmias, coronary artery disease (CAD), heart failure (HF), valvular disease, thromboembolic disorders, peripheral vascular disease (PVD), stroke, pulmonary hypertension, and pericardial complications (35–39, 45, 46). Anthracycline-induced cardiotoxicity occurs dose-dependently, with the highest incidence typically observed within the first year after chemotherapy completion (47–49). However, there is significant variation in patients’ susceptibility to anthracyclines (47–50). Approximately 9% of adult patients experience this cardiotoxicity, with the risk of HF related to doxorubicin estimated to have a cumulative incidence of 26% (51–53). Cisplatin, another commonly used chemotherapy agent, is linked to an increased risk of cardiovascular events even years after completing treatment (54, 55). In breast cancer survivors treated with trastuzumab for HER2 + breast cancer, studies predict a high incidence of cardiotoxicity, reaching up to 30% (56). The combination of trastuzumab and doxorubicin raises the risk of HF by more than 7-fold (57). Cardiotoxicity related to trastuzumab progressively increases during the 3–5 years after treatment completion and can persist for many years (58). Additionally, adjuvant hormonal therapy with tamoxifen elevates the risk of venous thromboembolism (59, 60). RT has been associated with various cardiovascular complications, affecting different components of the cardiovascular system, including the pericardium, myocardium, valves, coronary arteries, and conduction system. Asymptomatic breast cancer survivors who received RT show regional perfusion defects, closely linked to the left ventricle’s volume within the radiation field (61–67). The estimated incidence of RT-induced cardiotoxicity ranges from 10% to 30% at 5 to 10 years after treatment. Breast cancer survivors face an increased risk of thrombus formation, attributed to imbalances in von Willebrand factor release, thrombomodulin, and adenosine diphosphatase production, leading to heightened platelet adherence and clot formation in irradiated capillaries and arteries (68–72). A cross-sectional study using the National Health and Nutrition Examination Survey assessed the 10-year risk of atherosclerotic CVD using Pooled Cohort Equations in cancer survivors and non-cancer patients, revealing higher risk for cancer survivors compared to non-cancer patients (73). This increased prevalence and mortality have been attributed to cancer therapies, including RT and chemotherapeutic agents (74).

#### 1.1.3. Impact of aging on CVD associated with cancer treatments

As individual age, the prevalence of various disorders, including CVD, tends to increase within the population (75–79). Advanced age is a significant risk factor for doxorubicin-related cardiotoxicity, even at lower doses (80–82). CVD remains the leading cause of morbidity and mortality worldwide, particularly among individuals aged 65 and older. Scoring systems such as the Pooled Cohort Equations, Framingham Risk Score, and Reynolds Risk Score consistently highlight age as a significant risk factor for developing CVD, with both females and males facing an increased risk as they age (74, 83).

### 1.4. Cellular senescence in CVD associated with cancer treatments

#### 1.4.1. Cellular senescence—linking cancer, cancer treatments, and CVD

Cellular senescence, a hallmark of aging (84), plays a critical role in the development and progression of cardiac aging and CVD (74, 83, 85–87). Senescent cells accumulate in specific cardiovascular sites associated with CVD, such as atherosclerosis, HF, arterial stiffness, and hypertension (88, 89). Recent research has revealed that senescence is not limited to dividing cells due to replicative capacity loss (90–93). Instead, it is a broader cellular response to stress and damage, involving interconnected domains like DNA damage response (DDR), cell cycle arrest, senescence-associated secretory phenotype (SASP), senescence-associated mitochondrial dysfunction, autophagy/mitophagy dysfunction, nutrient and stress signaling, and epigenetic reprogramming. Activation of these domains occurs during senescence, with interactions between them. Cellular senescence significantly contributes to mammalian aging, playing a key role in age-related changes and dysfunction. Importantly, even post-mitotic cells, including cardiomyocytes, can undergo senescence (94, 95). Cardiac aging leads to various detrimental effects, including impaired angiogenesis (96), accelerated fibrosis (97), metabolic dysregulation (98), and the senescence and dysfunction of cardiomyocytes (99, 100). Senescent cardiomyocytes exhibit DNA damage, mitochondrial dysfunction, impaired contractile function, endoplasmic reticulum stress, hypertrophic growth, and secretion of SASP factors. Non-cardiomyocyte cells, such as fibroblasts, immune cells, and ECs, contribute to the regulation of cardiomyocyte senescence, further promoting cardiac aging and pathological remodeling (88, 101–104). Elevated activity of senescence-associated β-galactosidase (SA-β-gal), a widely used marker of cellular senescence, has been observed in ECs within atherosclerotic plaques in human coronary arteries (105).

The accumulation of senescent cells in the cardiovascular system during the aging process has been linked to age-associated disorders, including CVD (106–108). Senescent cells contribute to atherosclerotic plaque formation by secreting SASP factors, including proinflammatory cytokines and chemokines such as interleukin 1α, β (IL-1α, β), tumor necrosis factor α (TNFα), interferon γ (IFN γ), transforming growth factor β (TGFβ), and monocyte chemoattractant protein 1 (MCP-1). In early atherogenesis, oxidized low-density lipoprotein (oxLDL) accumulates in subendothelial spaces. SASP factors stimulates immune cell proliferation and activation, leading to monocyte recruitment into the subendothelial space. These monocytes differentiate into foam cell macrophages, contributing to plaque formation. Senescent ECs express vascular and intercellular cell-adhesion molecules (VCAM-1 and ICAM-1) and release cytokines, further attracting monocytes and promoting atherosclerotic plaque development (109–111). SASP factors also activate plasminogen activator inhibitor 1 (PAI-1), promoting thrombus formation (112). The persistent pro-inflammatory senescence phenotype induced by SASP factors leads to chronic inflammation, impairs cholesterol efflux from macrophages, and contributes to the accumulation of lipid-laden foam cells within the plaque (106–108).

Recent studies suggest cellular senescence’s involvement in plaque destabilization and rupture, leading to acute cardiovascular events. Senescent vascular smooth muscle cells (VSMCs) within the plaque show increased activity of matrix metalloproteinases (MMPs), contributing to plaque rupture and thrombosis (113). However, precise mechanisms connecting cellular senescence and CVD, such as chromatin structure remodeling, the local heart microenvironment, and the activation of DDR in cardiomyocytes leading to senescence, require further investigation.

Cellular senescence plays a dual role in cancer development and progression through the secretion of SASP factors (114). On one hand, it acts as a tumor-suppressor mechanism in various cancer treatments by inhibiting cell division and aiding in immune clearance of damaged cells, reducing the likelihood of tumorigenesis. However, cancer treatments inducing cellular senescence can have negative consequences, leading to persistent DNA damage, continued SASP factor secretion, and CVD development (115–122). SASP factors can induce inflammation, tissue remodeling, and angiogenesis, creating a microenvironment that supports cancer cell growth and spread (123, 124). The accumulation of senescent cells over time can lead to chronic inflammation, tissue dysfunction, and organ failure, increasing the risk of cancer development (125). Extensive research correlates senescent cell presence in tissues with an elevated cancer risk and poorer prognosis in cancer patients. Senescent cells can have both suppressive and promotive effects on cancer, depending on the context and specific cellular and molecular interactions involved.

#### 1.4.2. Mechanisms of cellular senescence: replicative senescence (RS) and stress-induced premature senescence (SIPS)

Cellular senescence plays a crucial role linking cancer, cancer treatments, and CVD, offering promise for preventing various CVD conditions such as acute myocardial infarction, atherosclerosis, cardiac aging, pressure overload-induced hypertrophy, heart regeneration, hypertension, and abdominal aortic aneurysm. Targeting cellular senescence is especially relevant for CVD prevention in cancer survivors. However, the mechanisms underlying cellular senescence are complex, as it can have both beneficial and detrimental effects in cancer and CVD, depending on the circumstances (126, 127). The pioneering work of Hayflick and Moorhead (1961) revealed that human diploid fibroblasts undergo irreversible cell cycle arrest after a certain number of divisions, known as the Hayflick limit (90–93). This arrest occurs due to telomere shortening, hindering the formation of T-loop (90–93) and leading to RS (128–130).

**Figure 1:**
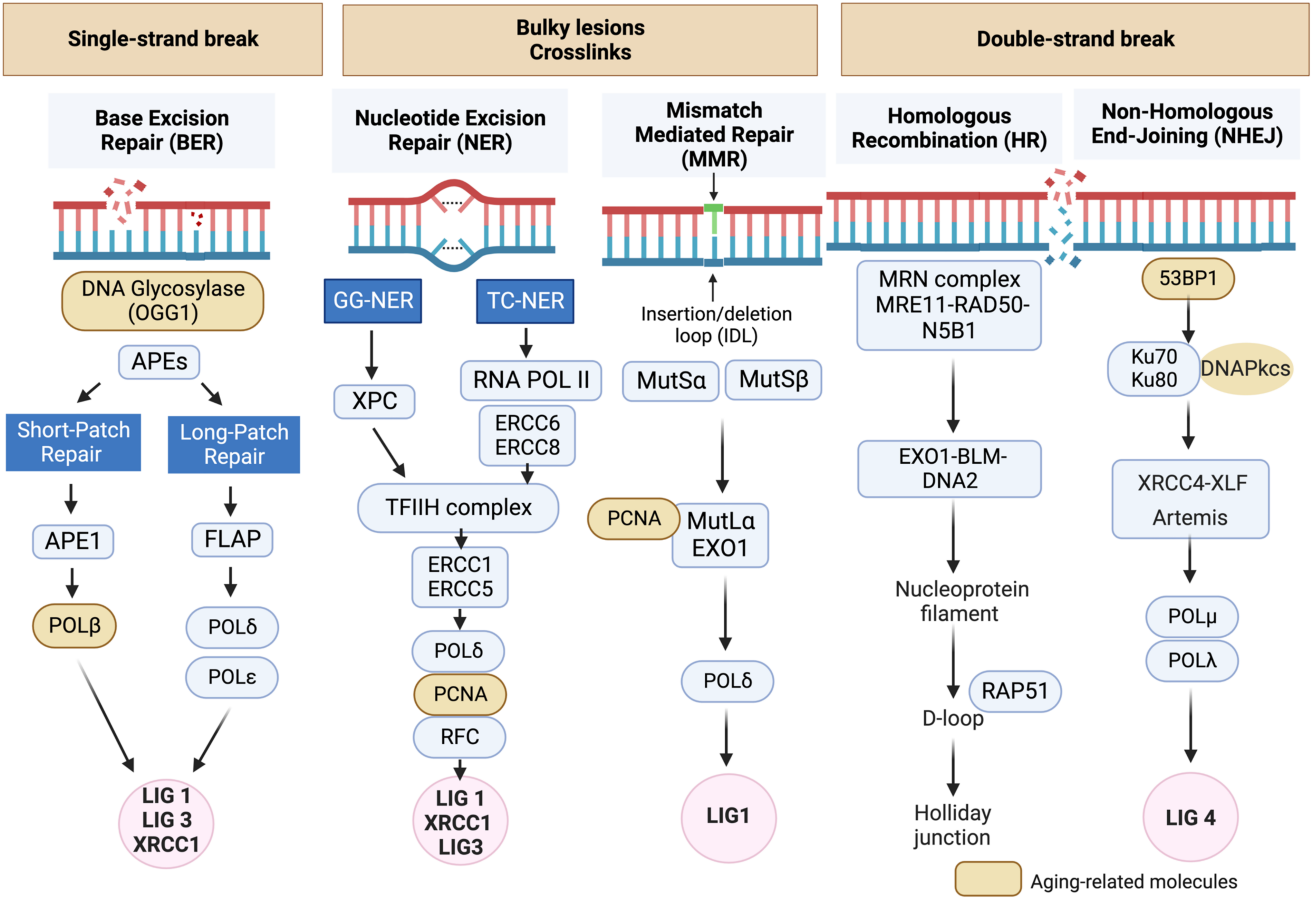
DNA damage response (DDR) mechanisms in aging-related disease: the DDR mechanisms depicted in this schematic are instrumental in preserving genomic stability and slowing down the aging process. However, as we grow older, the efficiency of these mechanisms may diminish, resulting in heightened accumulation of DNA damage and a decline in cellular function (134, 135). Schematic created using BioRender.com.

Cellular senescence occurs through RS over repeated replication cycles, leading to the exponential accumulation of senescent cells in multiple tissues with increasing age (131, 132). Alternatively, cellular senescence can be triggered prematurely by stressors, known as SIPS (133). These stressors can originate from internal and external factors, including oncogenes, RT, cancer treatments, mitochondrial dysfunction, reactive oxygen species (ROS), etc. (Figure 1). SIPS is characterized by events such as upregulated cyclin-dependent kinase inhibitors p21^Cip1/Waf1^, p16I^NK4a^, positive staining for SA-β-gal, and telomere shortening. Activation of the p53/p21^Cip1/Waf1^ and p16I^NK4a^/pRB pathways plays a central role in regulating senescence (132). Unlike RS, SIPS can occur without significant telomere shortening, as we (106, 116, 117) and others (132, 136) have previously reviewed. Senescent cells secrete SASP factors, including various cytokines, growth factors, and proteases contributing to chronic inflammation and senescence-associated phenotypes (115–122). In cardio-oncology, SIPS has emerged as a critical mechanism contributing to CVD development after cancer treatments.

#### 1.4.3. The central role of DNA damage and DDR pathways in cellular senescence

DNA damage can disrupt cellular homeostasis, leading to structural modifications that trigger cellular senescence during aging (137). Formation of γ-H2AX foci (γ-foci) indicates DNA damage is strongly associated with cellular senescence (11). DNA damage also induces mitochondrial ROS (mtROS) production and inflammation, further exacerbated by the mitochondria-nucleus feedback loop (115), as discussed in section (VI). Extrinsic factors such as free radicals, oxidizing agents, nitrosylating agents, alkylating agents, UV light, and certain cancer treatments like ionizing radiation (IR), can induce DNA damage (115, 137). In response to DNA damage, various DDR mechanisms are activated to recognize and repair different types of lesions, ensuring genomic integrity and DNA structure restoration. If the damage persists and cannot be effectively repaired, it can lead to RS (128–130), apoptosis, or SIPS (133) depending on the severity and nature of the damage and the cellular context (106, 116, 117, 132, 135–137). Cells utilize at least five distinct DDR mechanisms, including Based Excision Repair (BER) (138), Nucleotide excision repair (NER) (139, 140) and Mismatch repair (MMR) (141), each regulated differently to maintain genetic stability (Figure 1) (134, 135). Detailed mechanistic insights into these DDR mechanisms can be found elsewhere (134, 135, 137–140, 142–159).

#### 1.4.4. Mechanisms of cellular senescence regulation by DDR

Persistent activation of DDR pathways, crucial for cancer treatments, leads to the accumulation of inflammation, ultimately resulting in cellular senescence. DDR regulates cellular senescence and chronic sterile inflammation through various mechanisms. One mechanism involves the activation of DNA damage sensors that detect and respond to DNA damage signals. Another mechanism involves the alteration of the levels and activity of nicotinamide adenine dinucleotide (NAD^+^), an essential coenzyme involved in cellular metabolism and signaling pathways. DDR also influences mitochondrial function, linked to cellular senescence through ROS generation and metabolic changes. Furthermore, DDR modulates histone epigenetic changes and DNA modifications, leading to chromatin remodeling and alterations in gene expression patterns associated with cellular senescence (115, 137). Persistent activation of DDR also stimulates the secretion of SASP factors (115–122). For a comprehensive understanding of the mechanisms of cellular senescence regulation by DDR, each mechanism will be discussed in detail. As SASP factors play a critical role in CVD and cancer after cancer treatments, the discussion on SASP factors will be presented in a separate section (**IV**).

##### 1.4.4.1 Activation of DNA damage sensors

**Figure 2:**
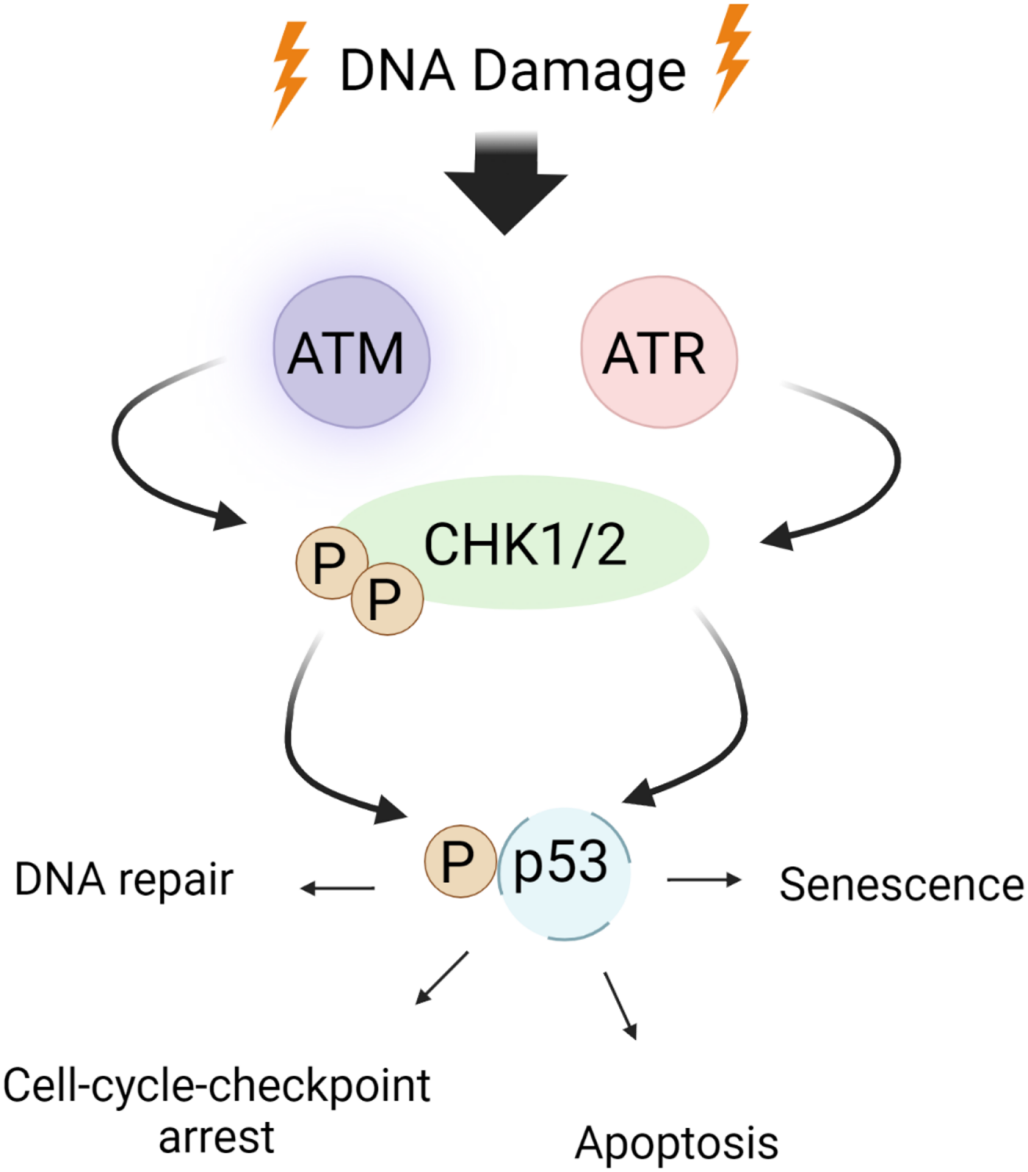
ATM/ATR signaling in the DDR: the DDR encompasses checkpoint kinases, downstream effector kinases, and a tumor suppressor protein, which collectively regulate apoptosis and cell cycle arrest. ATM/ATR, when activated by DNA damage, play critical roles in initiating the DDR. Upon activation, ATM/ATR phosphorylate various downstream targets, including CHK1/2. CHK1 phosphorylates p53 at S15 and Thr18 (160), thereby stabilizing p53 through prevention of p53 degradation and enhancing p53’s transcriptional activity. This ultimately leads to apoptosis and cell cycle arrest by inhibiting CDK activity. Schematic created using BioRender.com.

**Figure 3:**
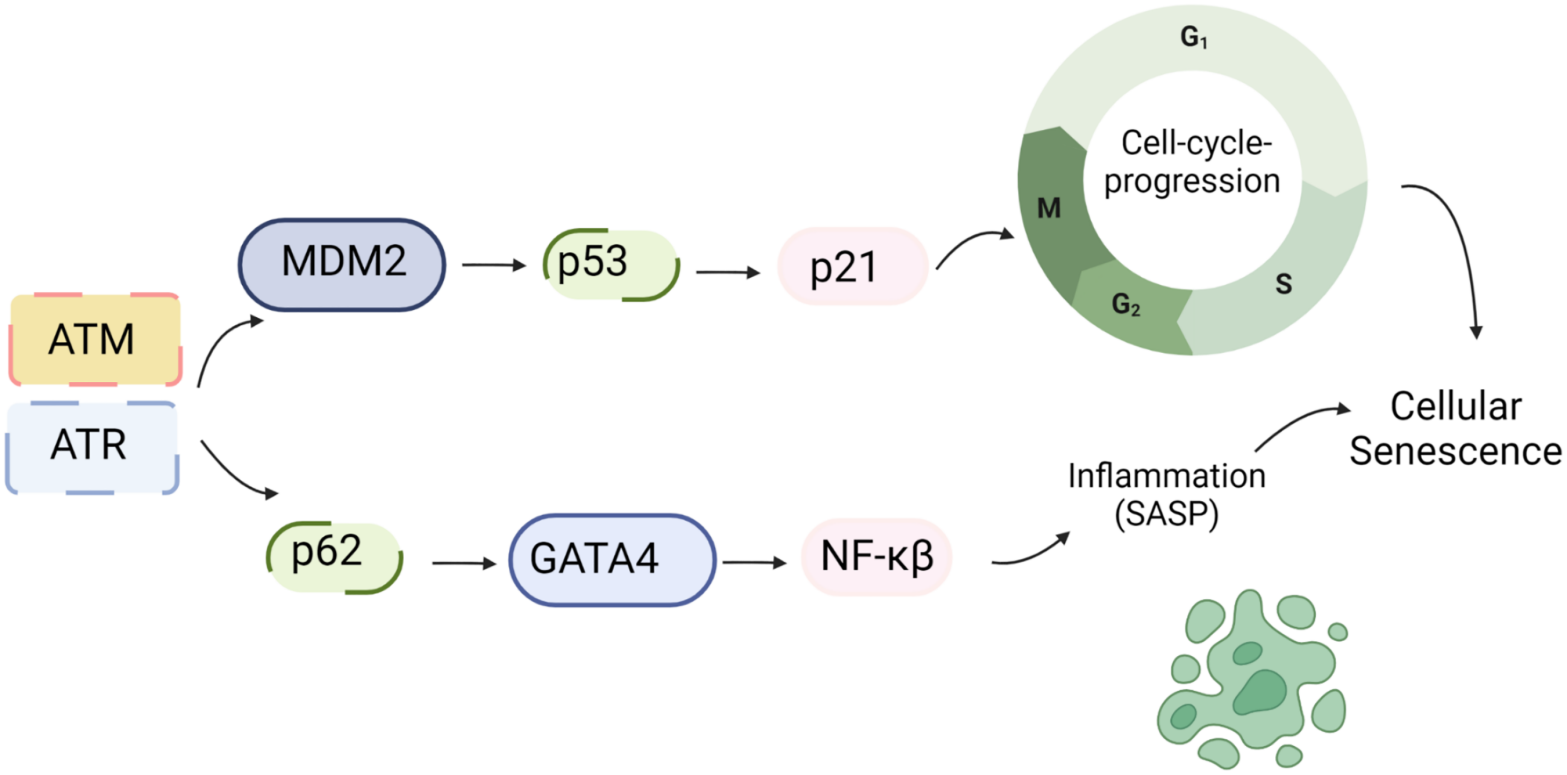
DDR and SASP: DNA damage triggers the activation of ATM/ATR, which subsequently phosphorylates CHK1/2. These phosphorylated kinases further activate p53. Upon activation, p53 transcriptionally upregulates p21, which inhibits CDK activity and leads to cell cycle arrest. Additionally, ATM/ATR can inhibit the interaction between p62 and GATA4, leading to the stabilization of GATA4. This stabilization event results in NF-kB activation and the subsequent induction of the SASP (161). Schematic created using BioRender.com.

DDR activates major DDR pathways such as ataxia-telangiectasia mutated (ATM) and ataxia-telangiectasia and Rad3-related (ATR) (Figures 2, 3). These sensors recognize DNA damage and initiate downstream phosphorylation events, involving checkpoint kinase 1/2 (CHK1/2) to coordinate the cellular response. Activated by ATM/ATR, p53 plays a critical role in responding to genotoxic stress, inducing apoptosis through targets like BAX and PUMA or promoting cell cycle arrest via p21^Cip1/Waf1^ and NOXA (115, 137, 163). p53 can inhibit NFκB activity, but its depletion may enhance tumor development by increasing inflammation (163–166). p53 also upregulates proinflammatory genes, such as IL6, CXCL1, and NFκB (167), with their secretion accelerated in senescent cells with high p53 activation (168, 169). In HF, inhibiting p53 can have cardioprotective effects by reducing cardiomyocyte apoptosis (170), but it may have atheroprone effects by increasing macrophage proliferation and/or inhibiting apoptosis, leading to larger plaques (171).

Higo et al.’s research reveals the association between HF and unrepaired single-strand breaks (SSBs), leading to DDR activation and inflammation by upregulating NF-κB signaling. Depletion of ATM attenuates pressure overload-induced HF, highlighting DDR’s causal role in HF (172). ATM/ATR can hinder p62-GATA4 interaction, inhibiting autophagy and ultimately activating SASP through NF-κB (161). While ATM/ATR activation can induce SASP, its impact on CVD, especially atherosclerosis, can be intricate due to additional effects on inhibiting macrophages, VSMC, and fibroblast proliferation (173, 174).

##### 1.4.4.2. Regulation of mitochondrial function

###### 1.4.4.2.1. Mitochondrial dysfunction, a hallmark of cellular senescence

**Figure 4:**
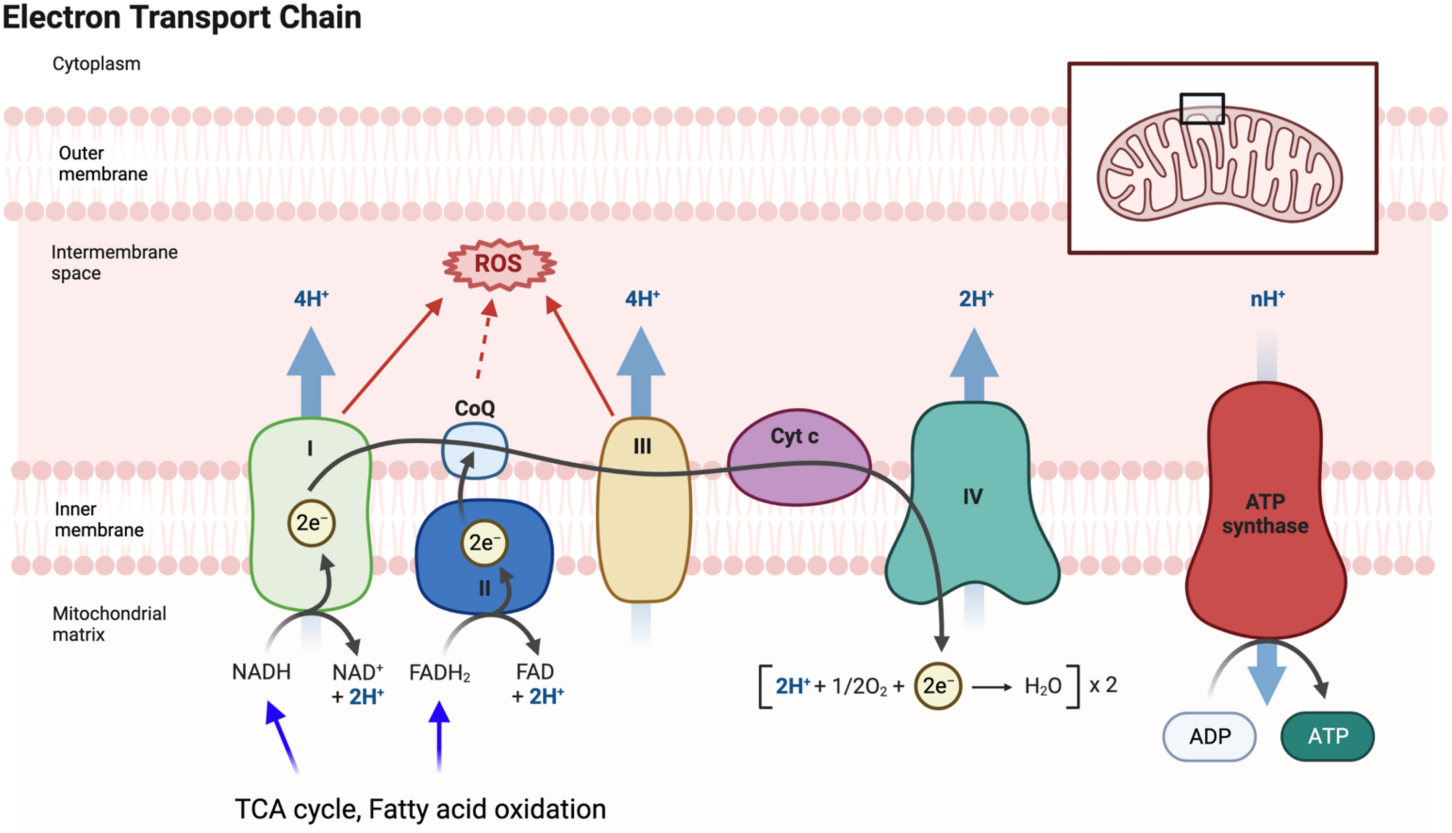
Electron transport chain (ETC) and mitochondrial ROS (mtROS) production: the ETC, located in the inner mitochondrial membrane, plays a vital role in the final steps of oxidative phosphorylation (OXPHOS). It facilitates the transfer of electrons from electron donors, such as NADH and FADH2, to oxygen, resulting in the production of ATP. As electrons flow through the ETC, protons are pumped across the membrane, creating a gradient that drives ATP synthesis by ATP synthase. During the electron transfer process in the ETC, there is also the generation of mtROS as a byproduct. These mtROS molecules can induce oxidative stress and cause damage to mitochondrial DNA, proteins, and lipids. FADH2 is a coenzyme involved in cellular respiration, and it donates electrons to the ETC. It is produced during the breakdown of fatty acids and certain amino acids. FADH2 specifically donates electrons to complex II of the ETC, bypassing complex I. This bypass leads to the production of fewer protons and consequently yields a lower amount of ATP compared to NADH. Figure generated using BioRender.com.

Mitochondrial dysfunction is a hallmark of aging (84), characterized by decreased respiratory capacity, membrane potential, and increased free radical production. Doxorubicin administration exacerbates damage to myocardial mitochondrial DNA (mtDNA) and activates mast cells, leading to harm to cardiomyocytes and impaired self-repair mechanisms (175). The interplay between aging and mitochondrial dysfunction contributes to a senescent phenotype (175, 176). The mitochondrial electron transport chain (ETC) uses mobile electron carriers (ubiquinone and cytochrome c) and four enzyme complexes (I, II, III, and IV) in the inner mitochondrial membrane (IMM) (Figure 4) (177, 178) to generate adenosine triphosphate (ATP) (179–184) through oxidative phosphorylation (OXPHOS) (177, 178, 185–187). During ETC activity, mtROS are produced (178), playing a crucial role in signaling pathways and maintaining cellular function (188). mtROS is also involved in the persistent SASP associated with cancer treatments (189). With aging, reduced antioxidant production increases ROS levels, leading to oxidation of lipids, proteins, and DNA (190, 191). ROS exposure can modify DNA’s guanine (G) to form 8-oxo guanine (8-oxoG), causing base-pairing errors during replication, resulting in genomic instability (192, 193), particularly in mtDNA due to less efficient repair mechanisms (194). Thus, mitochondrial dysfunction-mediated mtDNA damage contributes significantly to pathological conditions, including aging-related CVD.

###### 1.4.4.2.2. The balance of fusion and fission regulates mitochondrial function

**Figure 5:**
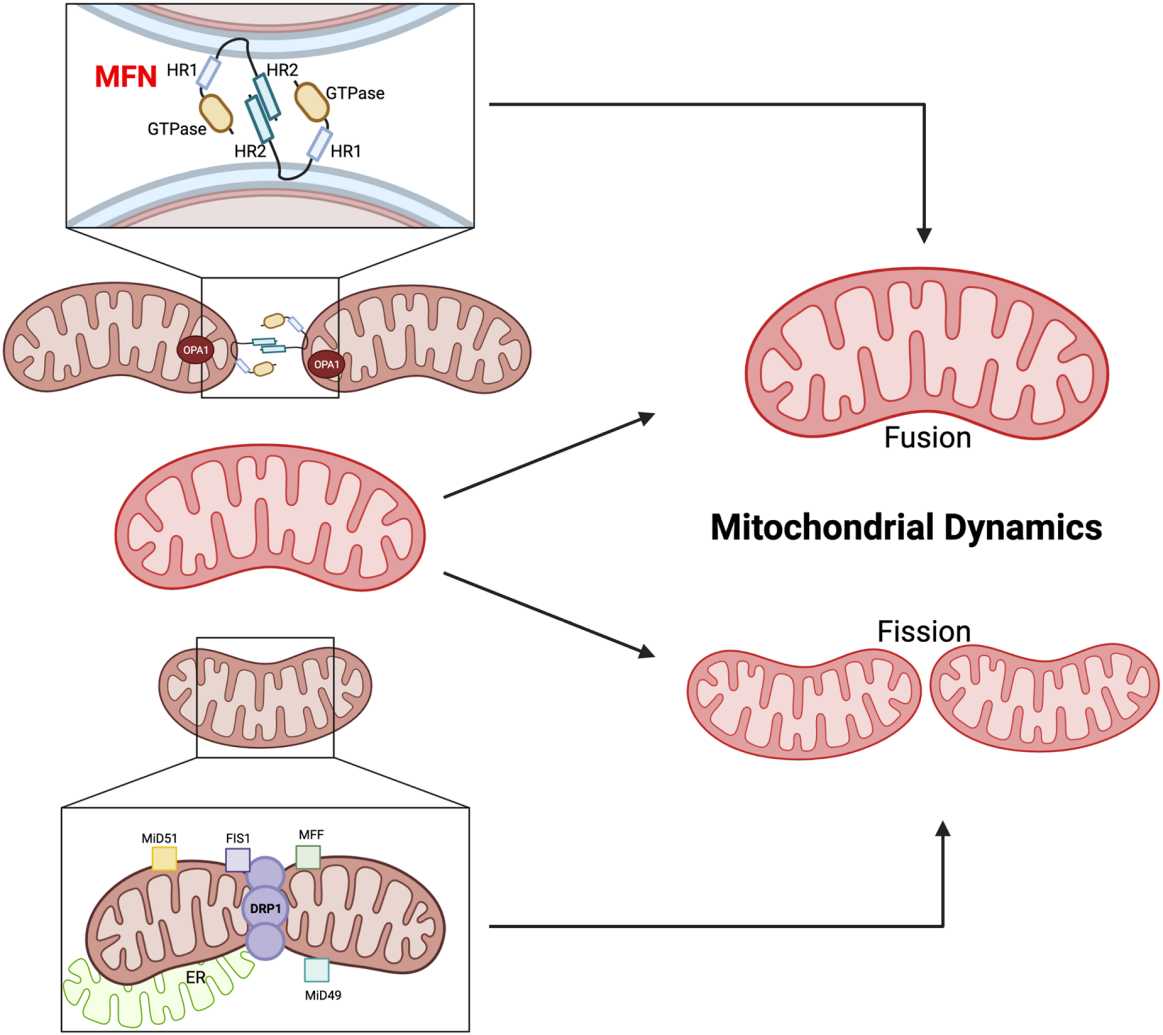
Mitochondrial fusion and fission. MFN1/2 and OPA1 are involved in OMM fusion, whereas OPA1 is involved in IMM fusion. The GTPase domain and Heptad repeat (HR) coiled-coil regions HR1/2 are shown. MFN2 interacts in transforming either homotypic or heterotypic (with MFN1) dimers to produce mitochondrial tethering and induces mitochondrial fusion. OPA1 binds the IMM. DRP1, MFF, Fis1, and the homologs MiD49 and MiD51 are involved in the fission process. MFF, Fis1, MiD49, and MiD51 act as receptors on the OMM to recruit and activate DRP1, which then oligomerizes and forms a ring-like structure around the mitochondrion at the site of division. The DRP1 ring generates tension, which causes constriction and eventual division of the mitochondrion into two daughter mitochondria (195). Figure created using BioRender.com.

**Figure 6:**
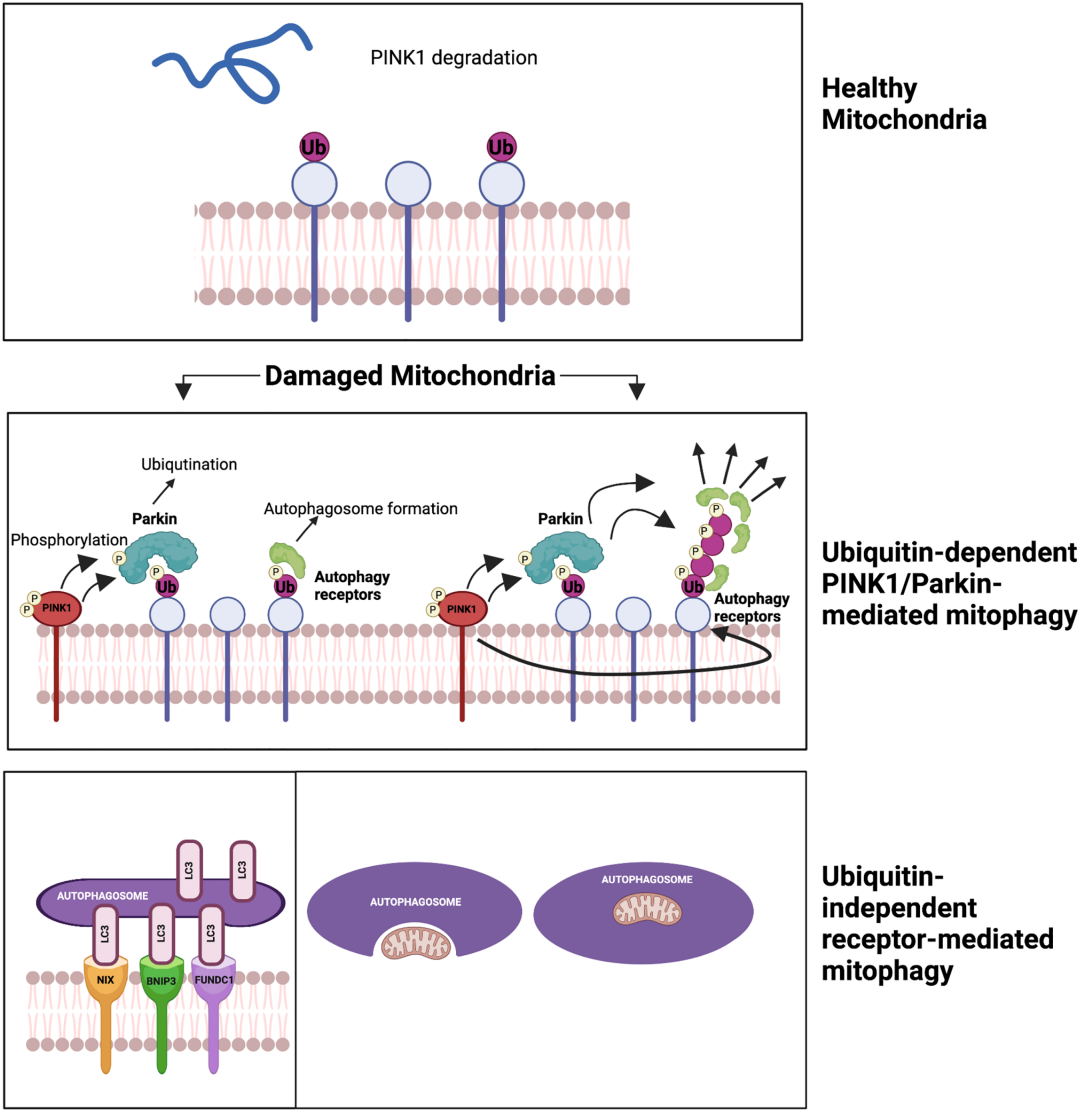
Two mechanisms of mitophagy (196) (please see the text) figure created using BioRender.com.

Mitochondrial dynamics, involving “fusion” (merging) and “fission” (separation) events, play a crucial role in maintaining mitochondrial health (Figures 5, 6). Mitofusin1/2 (MFN1/2) on the outer mitochondrial membrane (OMM) (197) facilitate fusion between adjacent mitochondria (198), regulated by dynamin-like GTPases (199). This process maintains inner membrane architecture through optic atrophy 1 (OPA1)-mediated fusion of the IMM (200). Conversely, mitochondrial fission is regulated by cytosolic GTPase dynamin-related protein 1 (DRP1) and mitochondria-bound proteins, such as mitochondrial fission factor (MFF), mitochondrial fission protein 1 (Fis1), and mitochondrial dynamics proteins of 51 and 49 kDa (MiD51 and MiD49). Defects in MFN1/2 or OPA1 can lead to mitochondrial dysfunction (201).

The balance between mitochondrial fusion and fission is critical for cellular homeostasis and preventing age-related pathologies. Perturbations in this balance can have significant consequences. Suppression of fission extends lifespan in Saccharomyces cerevisiae, while uncontrolled fission shortens lifespan (202). However, simultaneous suppression of both fission and fusion can lead to different effects on lifespan in yeast and worms (203). AMP-activated protein kinase (AMPK) and the target of rapamycin complex 1 (TORC1) are implicated in lifespan extension, with both fission and fusion playing roles in AMPK-induced longevity, while fusion appears particularly crucial for TORC1-mediated lifespan extension (204). These observations highlight the intricate involvement of mitochondrial dynamics in the aging process, but further research is needed to fully elucidate the precise roles of fission and fusion in aging and lifespan extension (120).

###### 1.4.4.2.3. The dysregulation of fusion and fission-related molecules is associated with cardiac aging

The dysregulation of fusion and fission-related molecules is associated with cardiac aging and diseases (205). DRP1 is crucial for mitochondrial fission and mitophagy (206). Cardiac-specific DRP1 knockout (KO) results in defective mitochondrial fission, increased mitophagy, mitochondrial loss, cardiomyocyte necrosis, and the development of dilated cardiomyopathy in mice (207–209). Conversely, cardiac-specific MFN1/2 KO leads to enhanced mitochondrial fragmentation, reduced mitophagy, mitochondrial accumulation, cardiomyocyte enlargement, progressive cardiac hypertrophy, and systolic dysfunction (210). Song et al. used cardiac-specific triple-KO mice lacking MFN1/2 and DRP1 to investigate the effects of eliminating both fission and fusion processes in the heart. The mice showed prolonged survival and developed pathological cardiac hypertrophy and HF resembling aged hearts. The researchers suggested that reduced fission and fusion processes leads to mitochondrial adynamism and senescence, impairing mitochondrial quantity control. These findings highlight how dysregulation of fission and fusion can contribute to cardiac dysfunction, hypertrophy, and aspects of aging in the heart (205).

###### 1.4.4.2.4. The impact of cancer and cancer treatments on fusion and fission

In cancer, proteins promoting mitochondrial fission are often upregulated in tumor tissues compared to normal tissues (211), and their increased expression is associated with worse prognosis for cancer patients (211). The global fragmentation of mitochondria in cancer cells leads to mtROS generation and metabolic alterations, promoting cancer cell migration, invasion, and metastasis (211). For certain cancers like pancreatic ductal adenocarcinoma (PDAC), abnormally fragmented mitochondria are linked to the oncogenicity of the disease. Therapeutic approaches targeting mitochondrial fusion restoration and normalizing morphology have shown promising results in preclinical PDAC models, reducing OXPHOS, inhibiting tumor growth, and improving overall survival (212).

Low-dose IR can increase mitochondrial fusion, which may inhibit tumorigenesis. However, dysregulation of both fission and fusion, leading to mitochondrial adynamism, can be detrimental to the cardiovascular system (205). Therefore, it is crucial to cautiously evaluate cancer therapies targeting mitochondrial fragmentation and their potential impact on the circulatory system. A thorough understanding of the interplay between mitochondrial dynamics, cancer biology, and cardiovascular health is crucial for developing effective therapies while minimizing potential cardiovascular complications associated with cancer treatments. Further research is needed to archive this comprehensive understanding.

###### 1.4.4.2.5. Cancer treatments and mitophagy

Mitochondrial autophagy, or “mitophagy,” was first described by John Lemasters (213). It selectively sequesters and encapsulates damaged or depolarized mitochondria into double-membraned autophagosomes, which then get degraded in lysosomes (214). There are two main mechanisms of mitophagy. The first mechanism is ubiquitin-dependent PINK1/Parkin-mediated mitophagy. It involves two key proteins, PTEN-induced putative kinase 1 (PINK1) and Parkin, recruited to damaged mitochondria. PINK1 phosphorylates ubiquitin, recruiting Parkin. Parkin then ubiquitinates mitochondrial proteins, forming ubiquitin chains signal the autophagic machinery. This triggers engulfment and degradation of the damaged mitochondria. PINK1’s phosphorylation of Parkin S65 is crucial for its activity and recruitment to the mitochondria, facilitating degradation through autophagy receptors (215, 216). The second mechanism is ubiquitin-independent receptor-mediated mitophagy, involving receptors on the OMM and not requiring ubiquitination of mitochondrial proteins. Autophagic receptors such as microtubule-associated protein light chain 3 (LC3), Bcl-2/adenovirus E1B 19-kDa interacting protein 3 (BNIP3), NIP-3-like protein X (NIX or BNIP3l), and FUN14 domain-containing protein 1 (FUNDC1) are involved in this pathway. These receptors possess LC3-interacting regions (LIRs) that bind to damaged mitochondria, promoting their engulfment and degradation by the autophagic machinery (217). This pathway plays a critical role in maintaining mitochondrial quality control and preventing the accumulation of damaged mitochondria, and its dysregulation can contribute to diseases, including CVD (218, 219).

Mitophagy, the removal of damaged mitochondria, reduces mtROS production and prevents apoptosis (220, 221). Nuclear and mitochondrial genes coordinate mitochondrial biogenesis (222), ensuring mitochondrial function and cellular homeostasis. The balance between mitophagy and mitochondrial biogenesis determines mitochondrial turnover, regulated by the mTOR signaling pathway. Activation of mTORC1 inhibits mitophagy, while inhibiting mTORC1 or activating FoxO1 restores mitophagy. mTORC1 activation is associated with mitochondrial protein aging and p62/SQSTM1-positive mitochondria accumulation, possibly contributing to aging (223). Reduced mitophagy leads to the accumulation of damaged mitochondria in aged C. elegans (224), while upregulation extends lifespan of nematodes (225). Impaired mitophagy is linked to aging-related diseases such as Parkinson’s, Alzheimer’s, and CVD (226–228). Interventions such as caloric restriction and exercise promote mitophagy and enhance lifespan (229, 230).

Parkinson’s disease is linked to disruptions in calcium (Ca^2+^) homeostasis and increased mtROS production (226). Depletion of PINK1 inhibits mitophagy, leading to excessive mitochondrial Ca^2+^ buildup in response to dopamine. Elevated Ca^2+^ levels promote mtROS production and trigger neuronal cell death in midbrain neurons (231). Accumulation of damaged mitochondria results in overproduction of mtROS and release of pro-inflammatory cytokines, contributing to neurodegeneration (232). Mitophagy plays a critical role in regulating mitochondrial quantity and quality, controlling mtROS production, mitigating mtDNA damage, and preventing cellular apoptosis. Impairments in mitophagy can lead to persistent immune system activation and the development of aging-related neurological disorders (214). Rita Levi-Montalcini and Barbara Booker (1960) observed that mature neurons acquire resistance to apoptosis. The following year, Leonard Hayflick and Paul Moorhead (1961) described “senescence,” a stable replicative arrest of cells *in vitro*. Neuroscience and senescence in cell biology developed independently for six decades, each unraveling molecular mediators and defining phenotypes related to their observations. Neuroscientists noticed neuron’s remarkable survival ability despite chronic inflammation and degeneration in their environment. Similarly, SIPS leads to a change in cell fate instead of responding to cellular or DNA damage through entering a stable cell cycle arrest. These cells secrete SASP factors, negatively impact the cellular environment. These fields of neuroscience and senescence have now intersected, with neuroscientists applying the concept of senescence to the brain, including post-mitotic cells. This integration is advancing the understanding of brain aging, neurodegenerative diseases, and CVD (233).

Mitophagy is observed in aged hearts and vessels (234, 235). However, its role in CVD and tissue damage remains unclear. The outcome of mitophagy upregulation in CVD conditions may depend on specific mitophagy levels. Excessive mitophagy and mitochondrial clearance can potentially harm the compromised circulatory system, as seen in HF and ischemic heart diseases (236). In cancer treatments, mitophagy activation enhances cytotoxic effects on cancer cells (237–239), while autophagy and mitophagy inhibitors can promote antitumor effects (240). Mitophagy’s role in cancer treatments may vary depending on specific mitophagy levels. The involvement of autophagy and mitophagy in cancer therapy-related premature aging requires further investigation.

###### 1.4.4.2.6. The opening of the mitochondrial permeability transition pore (mPTP) and its implications in aging-related CVD

Mitochondrial accumulation of Ca^2+^ and ROS can trigger the opening of the mPTP, releasing of pro-apoptotic proteins and cytochrome c into the cytosol (232). The ETC at the IMM establishes an electrochemical gradient, driving the uptake of mitochondrial Ca^2+^ from the sarcoplasmic reticulum. The Na^+^/Ca^2+^ antiporter (NCLX) expels Ca^2+^ from the mitochondria. Cytosolic Ca^2+^ overload leads to sustained increase in mitochondrial Ca^2+^ levels, triggering PTP opening. This results in loss of mitochondrial membrane potential, decreased ATP synthesis, OMM, disruption, and cell death (241, 242). While voltage-dependent anion channels (VDACs), adenine nucleotide translocase (ANT), mitochondrial ATP synthase (F0F1), phosphate carrier (PiC), and cyclophilin D (CypD) contribute to the mPTP, its exact structural configuration remains incompletely understood.

The opening of the mPTP is closely associated with the aging process. Upregulation of cyclophilin D (CypD) and its interaction with p53 triggers mPTP opening, while downregulation of heat shock protein 90 (HSP90) in aged cells increases mPTP opening. SIRT3, a key player in aging, inhibits mPTP opening by modulating CypD activity (243). The decline of SIRT3 during aging contributes to mPTP opening and impacts the regulation of aging. Treatments like metformin and caloric restriction, known for promoting longevity, prevent mPTP opening, potentially contributing to their beneficial effects on extending lifespan (244–246).

##### 1.4.4.3. Regulation of epigenetics

Epigenetics refers to molecular modifications in chromatin (DNA and histone proteins) that regulate gene expression without altering the DNA sequence (247, 248). These modifications can be influenced by factors like the environment, diet, social conditions, and cancer treatments, potentially passing down to future generations (249). Epigenetics plays a crucial role in the development of heritable (248) and are implicated in various diseases, including cancer, autoimmune, degenerative, neuroendocrine, neuropsychiatric, and CVD (247, 249). Certain epigenetic alterations like histone modification and DNA methylation can contribute to age-related diseases such as cancer and CV (249–252).

###### 1.4.4.3.1. DNA methylation

One extensively studied chromatin modifications is the methylation of cytosine residues at carbon 5 (5 mC) within CpG dinucleotides. This modification plays a crucial role in gene expression regulation and is linked to carcinogenesis. Three DNA methyltransferases (DNMTs) have been identified: DNMT1, DNMT3a, and DNMT3b. DNMT1 maintains DNA methylation patterns by methylating hemimethylated DNA during replication. DNMT3a and DNMT3b act as *de novo* methyltransferases, establishing DNA methylation patterns, and coordinating chromatin templating processes. During embryonic development, DNMT3a and DNMT3b exhibit significant *de novo* methyltransferase activity (247).

Adverse alterations in DNA methylation can lead to genetic abnormalities, potentially causing cancer. These modifications may silence tumor suppressor genes or activate oncogenes. Different types of cancer show specific aberrations in DNA methylation, such as hypermethylation or hypomethylation in critical DNA regions, as well as acetylation or methylation of histone proteins, especially within CpG islands. The nature of these modifications depends on the repressed tumor suppressor genes, regulating cell malignant growth (249, 250).

Aging is associated with genome-wide DNA methylation alterations (253, 254). Epigenetic drift lads to gradual epigenetic changes over time, causing variations in gene expression even among genetically identical individuals. As twins age, differences in methylation conversion rates and global DNA methylation patterns may arise (255, 256), potentially contributing to age-related diseases. Age predictors based on DNA methylation levels have been developed (257), like the DNA methylation PhenoAge clock, which assesses health and lifespan by evaluating specific CpG sites (258). However, the precise mechanisms underlying age-related DNA methylation changes and their role in the aging process and age-related diseases remain unclear (254).

###### 1.4.4.3.2. Histone modification

The nucleosome is a complex of four core histones (H2A, H2B, H3, and H4) wrapped around 147 base pairs of DNA, involved in DNA condensation and post-transcriptional modifications. Histone modifications include acetylation, methylation, ubiquitination, phosphorylation, and sumoylation (249).

Acetylation is a significant histone modification involving the addition of an acetyl group, affecting transcriptional pathways, DNA repair, and chromatin organization (247). Acetylation neutralizes the positive charge of lysine residues, weakening their interaction with histones and DNA. Two enzyme families, histone lysine acetyltransferases (HATs) and histone deacetylases (HDACs), dynamically and tightly regulate this process. HATs, including the CBP enzyme, have clinical relevance due to their role in neoplastic transformation and carcinogenesis (247, 250). Conversely, HDACs compact chromatin structure, repressing gene expression. HDAC inhibitors are used in cancer treatment to reactivate silenced tumor suppressor genes. HDACs are classified into four classes and can be involved in pathologies like leukemia through chimeric fusion proteins (247).

Aging is associated with alterations in histone modifications, particularly changes in acetylation and methylation patterns. Histone H4K16 acetylation, H4K20 trimethylation (H4K20me3), and H3K27 trimethylation (H3K27me3) are upregulated in aging (259). Nuclear SIRT deacetylation enzymes, such as SIRT1, SIRT6, and SIRT7 counteract aging effects. SIRT1 promotes chromatin silencing and transcriptional repression by deacetylating H1K26, H3K9, and H4K16 (260), while SIRT6 facilitates H3K9 and H3K56 deacetylation, contributing to the maintenance of genome stability and telomere function (261). Increased levels of H3K4 dimethylation (H3K4me2) are observed at stress response gene regions (262), and it plays a role in regulating hematopoietic stem cell identity and self-renewal abilities (263). Cells derived from progeria patients show an upregulation of repressive H4K29me3 (264, 265). The accumulation of repressive H3K27me3 in aged cells may lead to the permanent loss of transcriptional potential associated with aging.

HDAC inhibitors impact various hallmarks of aging, including epigenetic modifications, telomere attrition, genomic instability, loss of proteostasis, nutrient sensing dysregulation, mitochondrial dysfunction, cellular senescence, stem cell exhaustion, and intercellular communication (266). Trichostatin A, a selective inhibitor of HDAC class I and II, reduces pressure overload-induced cardiac hypertrophy by modulating relevant molecules (267). HDAC inhibitors like sodium butyrate has shown promise in inhibiting cardiac dysfunction in diabetic mice (268) and improving glucose metabolism in aged mice (269). These findings suggest that HDAC inhibition could be a potential therapeutic approach for cardiac diseases and may also have preventive effect against CVD by targeting the aging process.

##### 1.4.4.4. Regulation of autophagy

**Figure 7:**
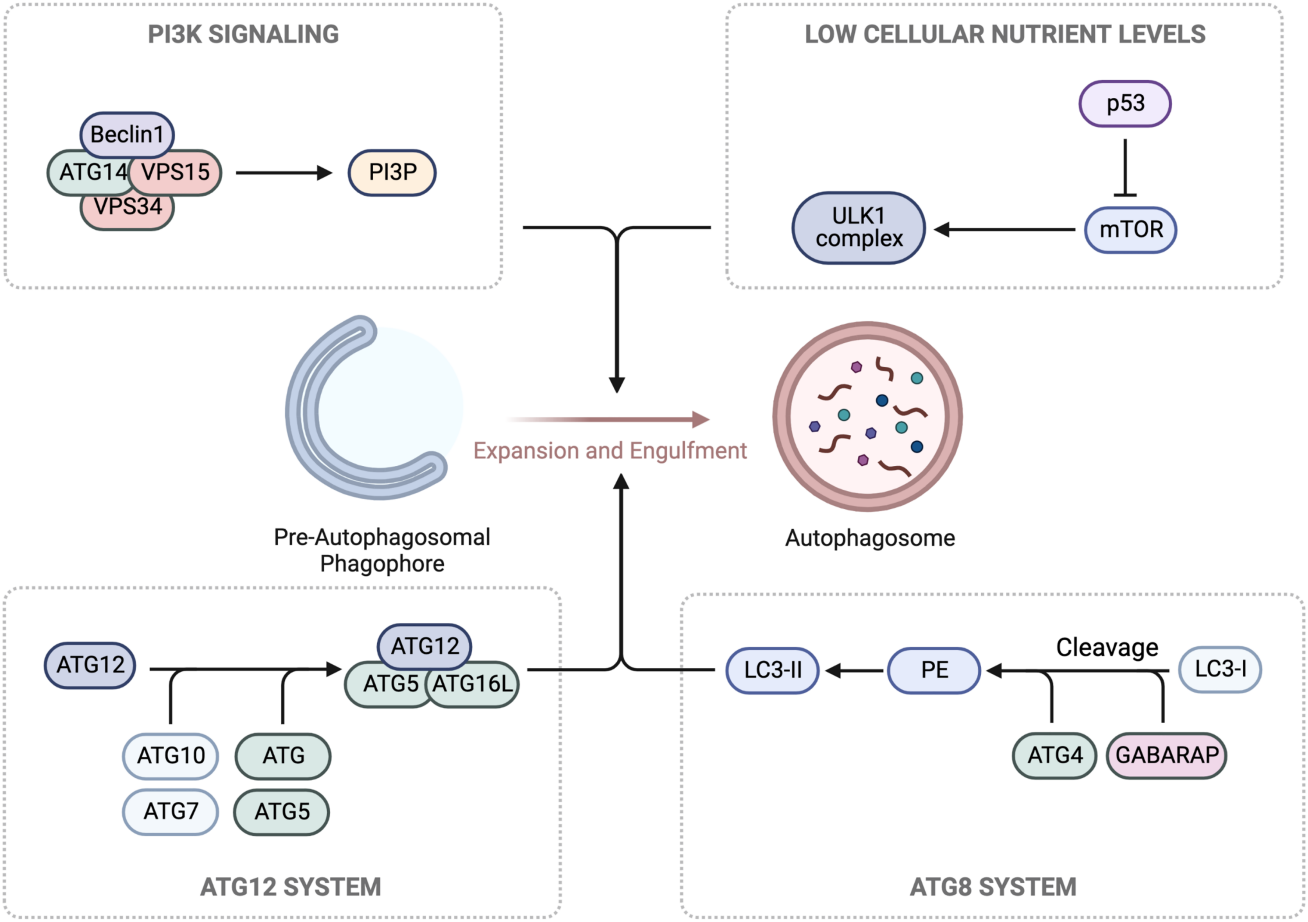
Autophagy machinery. In response to low glucose levels, AMPK pathways cause the phosphorylation and activation of the Unc-51-kinase 1 (ULK1) complex, which contributes to the growth of the pre-autophagosomal phagophore. Concurrently, ULK1 phosphorylation leads to the activation of the Class III PI3K complex, composed of Beclin1/ATG14/VPS15/VPS34, and the production of phosphatidylinositol 3-phosphate (PI3P) for further growth. For membrane binding and the final steps of autophagosome formation, conjugation of two ubiquitin-like complexes, ATG5/ATG12 and ATG10/ATG7, is necessary for the formation of the ATG5/ATG12/ATG16l complex. Hierarchically, this promotes the cleavage of LC3/GABARAP (LC3-I) by ATG4, generating phosphatidylethanolamine (PE), which forms LC3-II. Schematic created using BioRender.com.

Autophagy is a conserved process involving degradation of macromolecules and organelles within lysosomes (270, 271). It has three forms: macroautophagy (autophagy), microautophagy, and chaperone-mediated autophagy (CMA) (272). Autophagy sequesters targets within autophagosomes, which fuse with lysosomes for degradation. Microautophagy involves lysosome membrane invagination, while CMA uses chaperones to translocate unfolded proteins into lysosomes (273). The ULK1/ATG13/ATG101/FIP200 complex initiates autophagy in response to low cellular nutrient levels. The Beclin1/ATG14/VPS15/VPS34 Class III PI3K complex is recruited to autophagic cargo sites, generating phosphatidylinositol 3-phosphate (PI3P) for phagophore formation. ATG proteins are then recruited to facilitate membrane elongation and closure, forming a complete autophagosome. The ATG5/ATG12/ATG16l complex enables ATG8 (LC3 and GABARAP) conjugation to the membrane. The ATG5/ATG12/ATG16l complex converts ATG4-cleaved LC3/GABARAP (LC3-I) to LC3-II by conjugating it to phosphatidylethanolamine (PE), essential for autophagosome formation. The autophagosome fuses with a lysosome, forming an autolysosome for cargo is degradation and recycling (274) (Figure 7).

Autophagy plays a vital role in various fields, including cancer and degenerative diseases (270), but it also becomes critical in aging. The aging process involves the accumulation of damaged proteins and organelles, particularly challenging for non-proliferating cardiomyocytes (275, 276). As organisms age, declining autophagy levels hinder efficient cellular clearance, impacting heart tissue homeostasis. This downregulation of autophagy in aging hearts increases the risk of developing senescence-associated diseases (277). Autophagy is regulated by transcription factors like FoxO, HIF-1, p53, E2F1, NF*κ*B, KLF4, TFEB, and ZKSCAN3 (278), and in the aged heart, FoxO’s activity is inhibited, leading to reduced expression of autophagy-related molecules (278–280).

According to Levine and Klionsky, autophagy is a crucial component of the aging process in eukaryotic organisms, involved in the turnover of long-lived proteins and damaged organelles (281), Autophagy’s selective retention of components while eliminating senescent ones may slow down aging and age-related CVD. Autophagy has been observed in myocardial tissue cells, but its levels are relatively low. Conditions like congestive HF, CAD, hypertension, and aortic valvular disease can lead to the accumulation of autophagosome (282). CVD and stress can upregulate autophagy levels in the heart (283). Studies in ATG5-deficient mice have shown cardiomyopathy, hypertrophy, and contractile dysfunction, highlighting the significance of autophagy in maintaining cardiac function (284). However, it is important to note that increased autophagy levels have also been linked to HF and cardiomyocyte death through autophagy-induced degeneration (285). Thus, autophagy may have both beneficial and detrimental roles in the cardiovascular system.

##### 1.4.4.5. Regulation of NAD^+^

In its oxidized state, NAD^+^ regulates metabolic networks, including glycolysis, the TCA cycle, FAO, and NADH production (185, 286). Age-related studies show decreased NAD^+^ levels in senescent cells (287–290), linked to premature senescence and age-related diseases (189, 291, 292). Treatments like doxorubicin and IR activate poly (ADP-ribose) polymerase (PARP), deplete NAD^+^, and induce premature senescence (189). NAD^+^ supplementation has shown potential in counteracting aging effects (293, 294). NAD^+^ depletion during aging is associated with changes in circadian oscillation. NAD^+^ is a promising therapeutic target for promoting proper autophagy, delaying senescence, and addressing age-related diseases (295), making it a focal point in therapeutic development.

###### 1.4.4.5.1. Pathways of NAD^+^ biosynthesis

**Figure 8:**
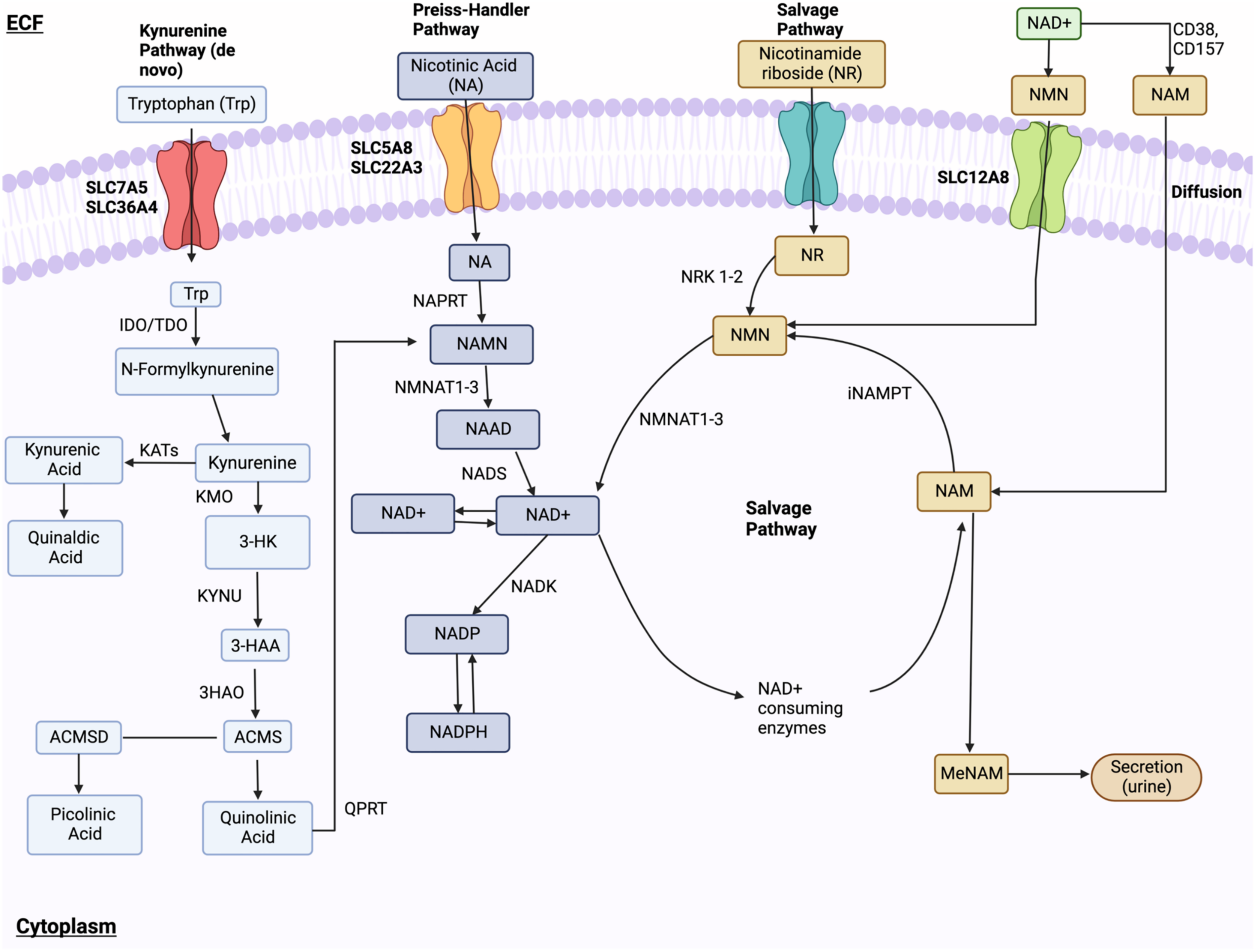
NAD**^+^** metabolism. NAD^+^ synthesis happens through the kynurenine (*de novo*) pathway, Preiss-Handler pathway, and NAD^+^ salvage pathway. In the kynurenine pathway, Tryptophan enters through receptors SLC7A5 and SLC36A4 and is changed into formylkynurenine, then kynurenine, and finally, kynurenic acid or 3-HK. These final compounds are further modified to 3-HAA. Subsequent modifications of 3-HAA into ACMS, quinolinic acid, NAMN, and NAAD lead to final NAD^+^ synthesis via NADS. In the Preiss-Handler pathway, NA is transported via SLC5A8 and SLC22A13 and is converted to NAMN. NR is brought into the cell and is modified into NMN, which is then ultimately converted to NAD^+^ by NMNAT1-3. Lastly, the salvage pathway recycles NAM produced by the NAD^+^-consuming pathway into NMN, which is ultimately converted into NAD^+^. Ectoenzymes CD38 and CD157 convert NAD^+^into NAM, which enters the cell and is converted into NMN. Lastly, NMN is dephosphorylated by CD73 into NR. Figure created using BioRender.com.

NAD^+^ synthesis involves three pathways: the kynurenine (*de novo*) pathway, Preiss-Handler pathway, and NAD^+^ salvage pathway (296). In the kynurenine pathway, tryptophan is converted to NAD^+^ via multiple enzymatic steps. This includes the conversion of tryptophan to kynurenine, then to nicotinamide mononucleotide (NAMN), and finally to NAD^+^. The Preiss-Handler pathway utilizes nicotinic acid (NA), converted to NAMN by NA phosphoribosyltransferase (NAPRT). In the salvage pathway, extracellular nicotinamide riboside (NR) or nicotinamide mononucleotide (NMN) can be converted to NAD^+^. NR is converted to NMN by nicotinamide riboside kinases (NRK) 1 and 2, and NMN is converted to NAD^+^ by NMNAT1-3. Nicotinamide (NAM) is recycled back to NAD^+^ via nicotinamide phosphoribosyltransferase (NAMPT) and CD73 (Figure 8).

###### 1.4.4.5.2. NAD^+^ metabolism in specific subcellular compartments

The NAD^+^ salvage pathway is vital for recycling and converting NAM into NAD^+^. It replenishes NAD^+^ levels after its degradation by NAD^+^-consuming enzymes, such as glycohydrolases (CD38, CD157), sterile alpha and toll/IL-1 receptor (TIR) motif-containing 1 (SARM1), protein deacylases (such as sirtuins (SIRTs), and PARPs. NAM, a by-product of NAD^+^ degradation (Figure 8), can inhibit NAD^+^ biosynthesis by feedback inhibition of NAMPT, the rate-limiting enzyme in the pathway. NAMPT’s expression tends to decrease during aging (297–300), potentially contributing to age-related NAD^+^ depletion. The salvage pathway maintains cellular NAD^+^ levels and counteracts NAD^+^ depletion, ensuring a continuous supply for cellular processes.

NAD^+^-consuming and generating enzymes are localized in different subcellular compartments, regulating NAD^+^ homeostasis. Intracellular NAMPT (iNAMPT) and NMNAT2 in the cytoplasm produce NAD^+^, while NMNAT3 and SIRT3-SIRT5 in mitochondria consume NAD^+^. NAMAT1 salvage nuclear NAD^+^. Shuttlers and transporters, like SLC25A51 and the malate/aspartate shuttle, maintain NAD^+^ levels between compartments. NAD^+^ is predominantly recycled is recycled through salvage pathways rather than being generated *de novo* (292, 301, 302).

###### 1.4.4.5.3. NAD^+^-consuming enzymes

NAD^+^-consuming enzymes, such as CD38, CD157, SARM1, SIRTs, and PARPs contribute to NAD^+^ depletion. Targeting the NAD^+^ salvage pathway and these enzymes may offer potential therapeutic approach to address aging and age-related diseases.

####### 1.4.4.5.3.1. CD38 and CD157

CD38 is expressed in various cell types and plays a role in tumorigenesis, aging, and disease. CD157, a paralogue of CD38, is primarily expressed in lymphoid tissue and the gut (303). They act as NAD^+^ hydrolases, generating NAM, cyclic-ADPR (cADPR), and nicotinic acid adenine dinucleotide phosphate (NAADP) from NAD^+^ degradation (304–306). Dysregulation of CD38 and CD157 is linked to diseases like Parkinson’s, ovarian cancer, and leukemia (307, 308). Increased CD38 and CD157 expression is observed during macrophage polarization and aging (288). CD38 KO mice show resistance to age-related NAD^+^ depletion and negative effects of a high-fat diet on NAD^+^ levels, liver health, and glucose metabolism (309, 310). CD38 overexpression reduces NAD^+^ levels and impairs mitochondrial function in mice (288, 310). CD38 activation can induce NAD^+^ depletion and EC dysfunction in the heart due to hypoxia-reoxygenation (311). CD38 inhibitors (thiazoloquin(az)olin(on)es and luteolinidin) prevent EC dysfunction and myocardial damage after ischemia (312, 313). Other CD38 inhibitors (apigenin and 78c) (314) show promise for treating aging-related diseases, including metabolic disorders and CVD (315).

####### 1.4.4.5.3.2. SARM1

SARM1 is a NAD^+^ cleavage enzyme found in neurons and other cell types (316–319) with two types of NADase activity. It hydrolyzes NAD^+^ to NAM and ADPR, and generates NAM and cADPR via ADP-ribosyl cyclase activity (317, 320). This depletion of cellular NAD^+^ levels and triggers axonal degeneration, which can be blocked by NAMPT or NMNAT overexpression and NR supplementation (321). SARM1 is crucial in regulating axonal degeneration after neural injury or disease (322). SARM1 KO rescues neuronal defects and embryonic lethality in NMNAT2 KO mice (323), indicating its NAD^+^-consuming role during embryonic development. SARM1 depletion also inhibits NMNAT2 deficiency-mediated axonopathy during aging (324). Notably, NAD^+^ inhibits SARM1 activity by competing with NMN in the presence of NMNAT2, and NMNAT2 depletion activates SARM1 (325, 326). SARM1 has been linked to age-dependent susceptibility to rotenone-induced neurotoxicity and may contribute to age-related neuronal loss (327). These findings provide insights into potential therapeutic targets for neurodegenerative diseases.

####### 1.4.4.5.3.3. Sirtuins (SIRTs)

Mammalian cells contain seven different SIRTs, major NAD^+^-consuming enzymes, localized in the nucleus (SIRT 1,6, and 7), cytosol (SIRT 1,2, and 5), and mitochondria (SIRT 3,4, and 5) (328). SIRTs remove acetyl group from lysine residues of proteins, leading to deacetylated lysin, NAM, and 2′-O-acetyl-ADP-ribose (329). SIRT levels decline during cellular senescence and aging (330). SIRT1 downregulates SASP factors like IL6 and IL8 via increased histone H3 and H4 acetylation (331). Inhibiting SIRT1 and SIRT6 causes premature senescence in ECs (332, 333). SIRT-activating compounds (STACs), including resveratrol, SRT1720, SRT3025, and SRT2104, are potential intervention for cardiomyopathy, metabolic syndrome, EC function, and atherosclerosis (334).

####### 1.4.4.5.3.4. Poly (ADP-ribose) polymerases (PARPs)

Seventeen isoforms with poly-(ADP-ribosyl) or mono (ADP-ribosyl) transferase activity for PARPs have been found, which consume NAD^+^ by transferring ADP-ribose to proteins, generating NAM. PARP1 acts as a sensor for DNA damage and is activated when DNA is damaged, leading to NAD^+^ depletion. Anti-melanoma cancer treatments activate PARP1-NF-kb signaling, resulting in SASP (335). However, excessive DNA damage can lead to PARP1 overactivation, causing cell death known as “Parthanatos” (336). PARP inhibitors such as olaparib, INO-1001 (an isoindolinone-based PARP inhibitor), and veliparib are used to treat CVD, pulmonary arterial hypertension, and cardiac repolarization in cancer survivors. PARP1 activation also plays a role in telomere length and telomerase activity maintenance during aging (337, 338).

##### 1.4.4.6. The interconnection between mechanisms regulated by DDR

Premature aging diseases like Xeroderma Pigmentosum, Cockayne syndrome, and Ataxia-telangiectasia may connect ROS generation, NAD^+^ depletion, autophagy, and mitophagy. Mitophagy loss leads to NAD^+^ depletion and mitochondrial dysfunction (339–342). Persistent DNA damage in these diseases activates PARP, depleting NAD^+^, and inhibiting NAD^+^-dependent SIRT activity and autophagy. Hyperactivated PARP can cause ATP depletion and cell death (343–346). Jiang et al. demonstrated that low H_2_O_2_ exposure induces PARP1 activation-mediated parthanatos, involving mitochondrial membrane potential decline, apoptosis-inducing factor translocation, and cell death. PARP1 activation inhibits autophagy, crucial for cell survival (347). Muñoz-Gámez et al. showed that PARP inhibition prevents ATP and NAD^+^ depletion, triggers autophagy via mTOR activation, and has cytoprotective effects (348).

Various interventions, including caloric restriction, intermittent fasting, genetic alterations (e.g., cardiomyocyte-specific dnPI3K or global AKT2 KO), and transgenic mouse models (e.g., cardiomyocyte-specific Parkin transgenic mice, SIRT1 transgenic mice, and pharmacological interventions like spermidine, rapamycin, resveratrol, and SRT1720), extend lifespan and delay aging in model organisms. These approaches activate cellular stress response pathways, enhancing maintenance and repair mechanisms, resulting in improved healthspan and longevity (349).

Recent research emphasizes the therapeutic potential of supplementing exogenous NAD^+^ precursors. This supplementation increases NAD biosynthesis, offering therapeutic advantages for various conditions, including metabolic, cardiac, and neurodegenerative disorders (334, 350). Elevated NAD^+^ levels improve mitochondrial function, stimulate SIRT-dependent mitochondrial recycling, and enhance expression and activity of autophagy/mitophagy-related molecules (294, 295, 339, 351–353). This leads to better organelle and protein aggregate clearance, interrupting the cycle of damage and NAD^+^ depletion. Boosting NAD^+^ levels can counteract depletion and support the resolution of physiological stresses, promoting long-term cellular health. NAD^+^ supplementation shows promise for addressing aging-related diseases and disorders.

### 1.5. Senescence-associated secretory phenotype (SASP)

#### 1.5.1. SASP has been implicated in CVD associated with cancer treatments

**Figure 9:**
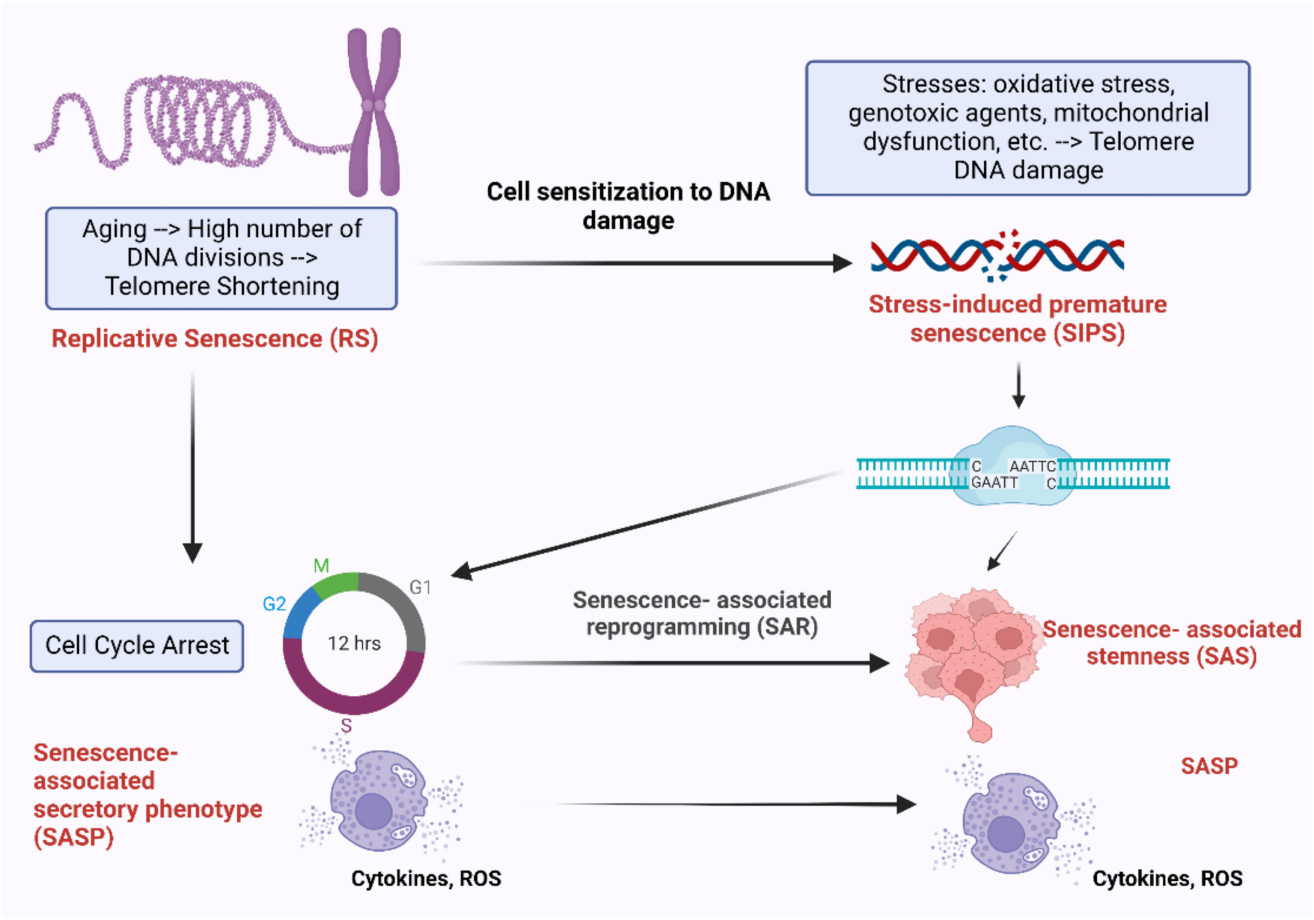
Different mechanisms of cellular senescence and their effects. During the process of aging, cells undergo irreversible cell cycle arrest after a certain number of divisions, RS. This process is accompanied by the progressive shortening of telomeres, the protective caps at the ends of chromosomes, which play a crucial role in cell division and survival (6–8). Cells experiencing RS enter cell cycle arrest and release pro-inflammatory cytokines, ROS, and other factors, known as SASP. Another form of senescence is SIPS, which is triggered by external factors such as oxidative stress, mitochondrial dysfunction, genotoxic agents, and others. Cells undergoing SIPS also enter cell cycle arrest and exhibit SASP. However, these senescent cells can bypass cell cycle arrest by undergoing senescence-associated reprogramming (SAR), leading to the development of SAS (68–70). Figure created using BioRender.com.

Senescent cells remain metabolically active, showing increased glycolytic activity (354, 355) and upregulated mtROS production and succinate induction, even with OXPHOS and glycolysis inhibition by low-dose IR, without undergoing necrosis or apoptosis (189). The SASP, consisting of soluble proteins and extracellular vesicles (EVs), includes pro-inflammatory cytokines, chemokines, growth factors, pro-angiogenic factors, small molecules, lipids (such as nitric oxide (NO) and prostaglandin E2 (PGE2)), ROS, and proteases (Figure 9) (356–358). Normally, SASP factors mediate communication between senescent cells and neighboring cells (106, 116, 117), facilitating immune cell recruitment for senescent cell removal. However, immunosenescence during aging (132, 136, 359) can compromise this clearance process, leading to the accumulation of senescent cells and their effects, contributing to CVD pathogenesis (360).

EVs, including exosomes and microvesicles, are small vesicles surrounded by a lipid bilayer membrane released by cells. They act as carriers of proteins, nucleic acids, and metabolites between cells, influencing the behavior of recipient cells (361, 362). Mesenchymal stem cell-derived EVs exhibit anti-inflammatory, anti-fibrotic, pro-proliferative, and pro-angiogenic properties through factors such as PGE2, tumor necrosis factor (TNF)-inducible gene 6 protein (TSG6), transforming growth factor β (TGFβ), interleukin 6 (IL6), interleukin-1 receptor antagonist (IL-1RA), NO produced by inducible NO synthase (iNOS), and kynurenine produced by indoleamine 2. 3-dioxygenase (IDO) (363–366). In contrast, activated senescent cells release more functional EVs than non-senescent cells due to upregulated p53 expression. These EVs can promote senescence in neighboring cells by enhancing ROS production (367, 368). EVs also contribute to cellular senescence by transporting senescence-inducing factors including miRNAs, long non-coding RNAs, and proteins that promote senescence in recipient cells (369).

#### 1.5.2. RT-induced immunosenescence via p90RSK activation

RT administered to the chest region increases CVD risk in patients and may cause mitochondrial dysfunction and cellular senescence (117). To investigate this, we studied 16 thoracic cancer patients who received RT. We analyzed peripheral blood mononuclear cells (PBMCs) before and three months after RT using mass cytometry (CyTOF) to characterize immune cell lineages and examine senescence, DDR, efferocytosis, and determinants of clonal hematopoiesis of indeterminate potential (CHIP).

Our analysis showed reduced B cell subtypes after RT. Clustering of CyTOF data revealed 138 functional PBMC subsets. Post-RT samples displayed increased TBX21 (T-bet) expression in the largest subset of Ki67–/DNMT3a + naive B cells, with T-bet expression correlated with p90RSK phosphorylation. CD38 expression was elevated in naive B cells (CD27–) and CD8 + effector memory CD45RA T cells (TEMRA). In vitro experiments confirmed the crucial role of p90RSK activation in upregulating CD38+/T-bet + memory and naive B cells, myeloid cells, β-gal staining, and mitochondrial ROS following radiation exposure. These findings indicate the pivotal role of p90RSK activation in immunosenescence (370).

Previous studies emphasized the critical role of p90RSK activation in immune cells and its involvement in the development of atherosclerosis through T-bet induction. T-bet directly binds to the CD38 promoter region, resulting in increased CD38 expression. Given the significant roles played by both T-bet and CD38 in immunosenescence, our data provide insights into the cellular and molecular mechanisms linking RT-induced p90RSK activation, immunosenescence, T-bet, and CD38 induction observed in thoracic cancer patients undergoing RT. Targeting the p90RSK/T-bet/CD38 pathway may prevent RT-associated CVD and improve cancer prognosis by inhibiting immunosenescence (370).

#### 1.5.3. Senescence-associated stemness (SAS)

Senescence serves as a tumor-suppressor mechanism, but key regulators of senescence, such as p16^INK4a^, p21^CIP1^, p53, and H3K9me3, also impact stem cell functions, or “stemness.” Recent studies have shown that senescence can drive cancer cells towards stemness, promoting cancer stem cell regeneration (114, 371–376). Acquiring stemness properties in cancer cells can influence tumor aggressiveness and clinical outcomes. Milanovic et al. analyzed senescent and non-senescent B-cell lymphomas in mice (374), finding that senescent cells upregulated adult tissue stem cell signature, activated Wnt signaling, and distinct stem cell markers. Releasing cells from senescence led to enhanced clonogenic growth potential and higher tumor initiation potential *in vivo*, dependent on Wnt signaling. Inducing senescence in leukemia models reprogrammed non-stem bulk leukemia cells into self-renewing, leukemia-initiating stem cells. Human cancer cell lines and primary samples of hematological malignancies confirmed these findings. Milanovic et al. revealed that certain senescent cancer cells undergo reprogramming in response to chemotherapy, acquiring a proliferative phenotype known as SAS (374). In this state, they bypass senescence-induced cell cycle arrest and exhibit heightened clonogenic growth potential (374–376). SAS is regulated independently of cell cycle arrest (377, 378) and cell death (132, 335, 374, 375, 377–385). This adaptive mechanism can contribute to cancer cells developing resistance against chemotherapy and RT, leading to survival and continued proliferation despite treatment (379, 380, 382)*.* These findings highlight the role of senescent cancer cells in treatment resistance and tumorigenesis, even in cancer survivors (371, 372). Additionally, SAS may also occur in vascular cells, including ECs and myeloid cells. Considering the contributions of both senescence and macrophage proliferation to CVD (386), the SAS phenotype in vascular cells may significantly impact the development and progression of CVD.

#### 1.5.4. The role of the macrophage ERK5-NRF2 axis in SASP

Extracellular signal-regulated kinase 5 (ERK5) is a protein with dual kinase-transcriptional activity (387–392). Inhibitors targeting ERK5's catalytic activity have been evaluated for cancer and inflammatory disease treatment (388, 393). However, recent evidence questions the significance of ERK5's catalytic activity in proliferation and inflammation. Our recent investigation aimed to understand how ERK5 drives the proinflammatory senescent phenotype in myeloid cells, contributing to atherosclerosis (394). We generated ERK5 S496A knock-in (KI) mice using CRISPR/Cas9 technology and induced hypercholesterolemia for atherosclerosis characterization. Plaque phenotyping was performed using imaging mass cytometry in both homozygous ERK5 S496A KI and wild-type (WT) mice. We also conducted RNA sequencing and *in vitro* assays, including senescence, mitochondrial ROS, inflammation assays, and metabolic extracellular flux analysis using bone marrow-derived macrophages from hypercholesterolemic mice. Our data analysis demonstrates that ERK5 S496A KI mice show inhibited atherosclerosis. We observed that ERK5 S496 phosphorylation mediates SASP and SAS through upregulation of the aryl hydrocarbon receptor (AHR) in plaques and macrophages from hypercholesterolemic mice. ERK5 S496 phosphorylation induces SUMOylation of NFE2-related factor 2 (NRF2) at K518, inhibiting NRF2’s transcriptional activity, without affecting ERK5's catalytic activity. Specific ERK5 kinase inhibitors (AX15836 and XMD8-92) can inhibit ERK5 S496 phosphorylation, suggesting that ERK5 S496 phosphorylation is involved in the anti-inflammatory effects of these inhibitors. Our study uncovers a novel mechanism involving the macrophage ERK5-NRF2 axis, significantly contributing to the SAS via AHR upregulation. This mechanism explains the paradoxical presence of senescence in proliferative plaques, allowing myeloid cells to bypass senescence-induced cell cycle arrest during atherosclerosis formation. Furthermore, the SAS phenotype provides valuable insights into the dynamics of cellular senescence within atherosclerotic lesions. These findings advance our understanding of atherogenesis at the molecular level and offer potential therapeutic strategies targeting the SAS phenotype (394).

### 1.6. Rethinking the relationship between telomere shortening and cellular senescence

#### 1.6.1. Telomere DNA damage results in the persistent activation of DDR pathways

Nakamura et al. made an important discovery, revealing the presence of γ-foci at uncapped telomeres and non-telomeric DNA damage sites in both humans and mice, suggesting that both types of DNA damage contribute to cellular senescence (11).

Telomeres, composed of repetitive DNA hexanucleotide TTAGGG sequences, are situated at the ends of mammalian chromosomes (395). They play a crucial role in protecting the linear chromosome structure. When unprotected, chromosome ends can resemble damaged DNA, triggering a DDR that leads to cell cycle arrest through p53-mediated p21 upregulation, ultimately resulting in cellular senescence (396). Telomere shortening occurs over multiple cell divisions, reaching the Hayflick limit, leading to RS and limited cell replication (90–93). Thus, telomere shortening serves as a marker of cellular aging and limited replicative capacity.

Recent studies challenge the direct correlation between telomere shortening and cellular senescence in non-dividing, quiescent, and terminally differentiated cells like cardiomyocytes. The link between telomere length, cardiac hypertrophy, fibrosis, and age-related cardiac dysfunction remains uncertain (397). Both human and murine cardiomyocytes show a senescent-like phenotype characterized by persistent DNA damage at telomere regions during aging, independent of telomere length and cell division, driven by mitochondrial dysfunction. This telomere damage activates senescence-inducing pathways (p21^Cip1/Waf1^ and p16I^NK4a^) leading to a non-canonical SASP, contributing to age-related cardiac fibrosis and myocardial hypertrophy (77). Interestingly, cellular senescence can still occur with telomerase expression, as observed in immortalized human foreskin fibroblasts expressing telomerase (hTERT-BJ1) exposed to stressors like hydrogen peroxide or Ultraviolet B radiation (91). Renal tubular cells exposed to urine from patients with calcium oxalate kidney stones can undergo SIPS due to oxidative stress from oxalate and calcium oxalate monohydrate (398). Fairlie and Harrington’s study revealed that cell clonogenic survival following IR was dosage-dependent and increased when telomere lengths exceeded 17 kbp, even with telomeric DNA present in all chromosome ends (399). Post-mitotic cells like cardiomyocytes and neurons, which have longer telomeres, can experience persistent telomere DNA damage, leading to telomere-associated DDR foci (TAFs) in aging (77, 111, 400). In models of vascular aging, human telomerase overexpression restored telomere length and the shelterin complex expression, reducing DNA damage and its effects (401–403). Telomerase has broader effects on aging beyond telomere maintenance, including decreased secretion of inflammatory cytokines, normalization of cell and nuclear morphology, restoration of replicative capacity and cell functions (e.g., angiogenic processes), modulation of epigenetic marks, and adjustments to the transcriptional profile. In a murine model of Hutchison-Gilford Progeria syndrome, murine telomerase expression reversed vascular senescence and extended lifespan (403). Studies show that senescence can be induced in mouse embryonic fibroblasts exposed to 20% oxygen density, independent of concurrent telomere shortening (18). These findings suggest that telomere length alone may not be directly linked to the aging process, particularly in non-dividing, quiescent, rarely dividing/post-mitotic cardiomyocytes, or terminally differentiated cells, and that telomere shortening-independent pathways of senescence exist. These findings emphasize the critical role of telomere DNA damage and the resulting persistent activation of DDR. Telomere dysfunction, rather than telomere length alone, may be a more reliable indicator of SIPS. necessitating the evaluation of both factors for a comprehensive telomere status assessment and cellular age (399, 404–408). Various methods are available for measuring telomere length (409). Terminal restriction fragment (TRF) analysis involves Southern blotting and estimation of intensity and size distribution (410), while quantitative PCR-based measurement assesses the telomere-to-gene signal ratio (411, 412). In situ hybridization (Q-FISH) uses quantitative fluorescence on dividing cells (413–415), and single telomere length analysis (STELA) allows high-resolution measurements (416). The telomere short length assay (TeSLA) offers a more sensitive, efficient, and specific approach for detecting telomere length compared to other techniques.

Telomere DNA, rich in guanine, is susceptible to oxidation due to guanine’s low redox potential (417). Repair mechanisms for telomere DNA damage are less efficient compared to genomic DNA repair (400), and the repair process is significantly slower, taking up to 24 h (418, 419). This results in persistent DNA damage at telomere, leading to the continuous activation of DDR (397, 420). Research by Benetos et al. suggests that telomere attrition, rather than inherently short leukocyte telomere length at birth, primary contributes to shorter telomere length in atherosclerotic CVD patients (421). Telomere-binding proteins, such as Shelterin components, play a crucial role in preventing DNA damage (422, 423), and their dysfunction leads to the persistent activation of DDR at telomeres and the formation of TAFs (92, 111, 420, 424).

#### 1.6.2. Mechanisms of telomere dysfunction

Telomere dysfunction arises from disruption in the Shelterin complex, composed of TRF1, TRF2, POT1, TIN2, TPP1, and TERF2IP (or RAP1). Shelterin plays a vital role in maintaining telomere length and preventing shortening (425). TRF1 and TRF2 bind to double-stranded telomeric DNA as homodimers, facilitated by the TRFH domain for dimerization and DNA binding (426). TIN2 associates with TRF1 and TRF2, stabilizes TRF1 and TRF2 at telomeres (427) and interacts with TPP1, which is essential for proper telomeric localization of TPP1 and POT1, ensuring telomere integrity (396, 428, 429).

ATM/ATR kinases are key regulators of DDR signaling pathways. They phosphorylate proteins at DNA damage sites, such as histone H2A (H2AX), forming phosphorylated γ-foci. CHK2 and CHK1 are also activated by ATM/ATR (430), potentially leading to cellular senescence (425). TRF2 suppresses the ATM pathway during DSBs, and POT1 suppresses the ATR pathway during SSBs (428), suggesting Shelterin prevents improper DDR activation and telomere dysfunction without affecting telomere length (431).

Adult tissues typically exhibit minimal or absent telomerase activity, suggesting that a decrease in the levels of Shelterin components can result in telomere shortening and cellular senescence (432). Telomeric DNA is susceptible to oxidation, leading to telomeric DNA oxidative damage and reducing the amount of bound TRF1 and TRF2 in vascular cells (417). Conversely, TRF2 overexpression in human fibroblasts reduces oxidative damage to telomeric DNA (433). In patients with CAD, there is an increased presence of senescent ECs with lower TRF1 levels and greater telomeric DNA oxidative damage (434). Serial passaging of human umbilical vein ECs, a cell culture model of aging, leads to decreased TRF1 expression (435). However, TRF1 overexpression in this model decreases TAFs (435) and SASP (436). This decrease in bound TRF1 and TRF2 may explain vascular cell senescence under oxidative stress. Studies on ECs and VSMCs reveal a connection between oxidative stress-induced telomere dysfunction and Shelterin protein expression. Overexpressing TRF2 in VSMCs reduces DNA damage and enhances DNA repair, highlighting the regulatory role of Shelterin proteins in oxidative stress-induced telomere dysfunction (437). Telomere dysfunction and SASP precede RS, potentially contributing to vascular dysfunction and the development of CVD by promoting early changes that lead to senescence in ECs and VSMCs (436). Preserving the structure and function of Shelterin presents an appealing strategy to control or prevent cellular senescence-associated CVD.

#### 1.6.3. Posttranslational modifications (PTMs) of TERF2IP in atherosclerosis

**Figure 10:**
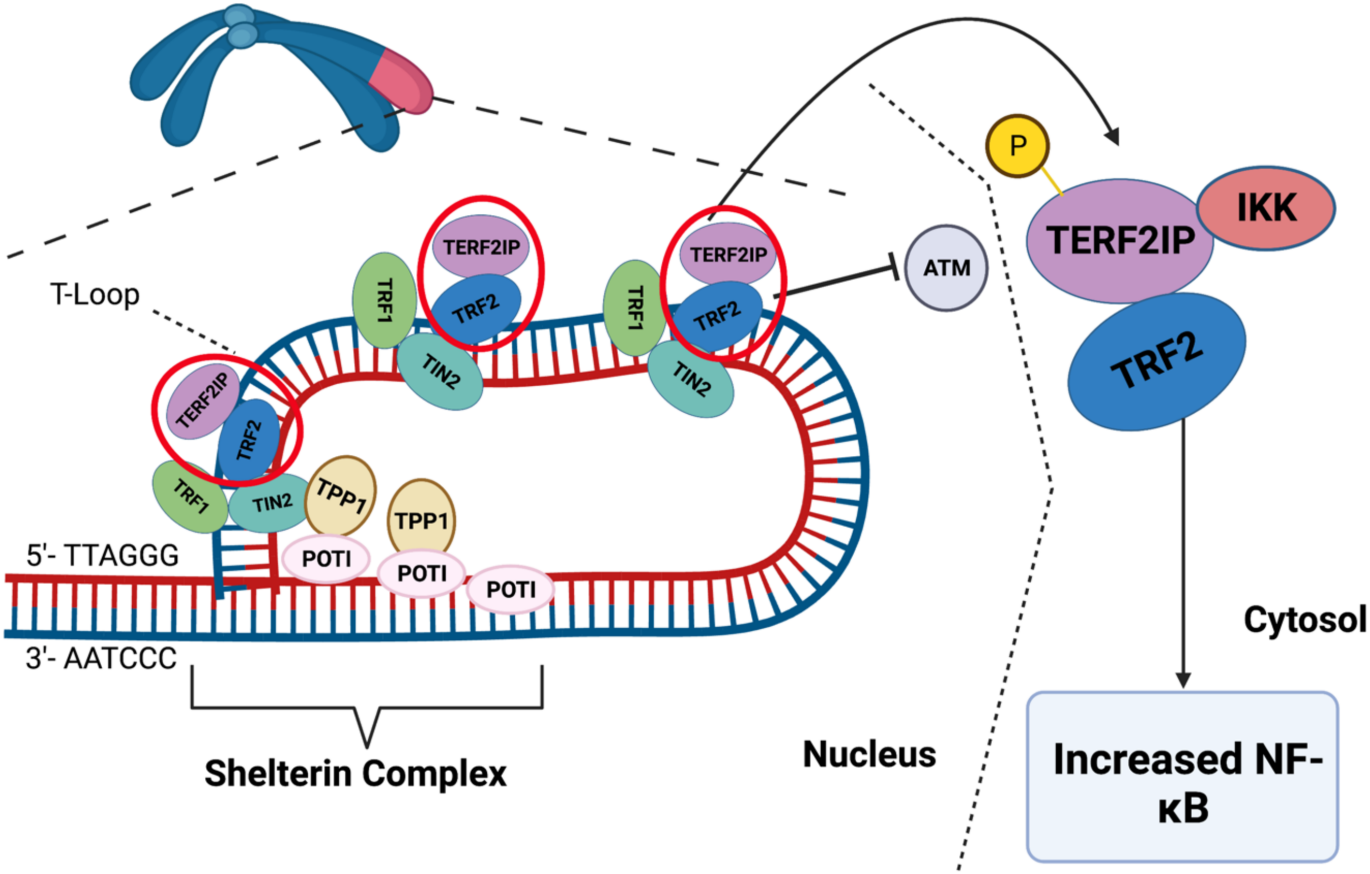
Scheme of Shelterin complex and t-loop. The ends of chromosomes have non-coding sequences called telomeres to protect the ends of DNA strands. The Shelterin complex is a group of proteins that maintain the T-loop, which protects the telomere ends from activating DDR. The Shelterin complex is made up of six proteins: TRF1, TRF2, POT1, TIN2, TERF2IP, and TPP1. These proteins can regulate telomere length, prevent telomere DNA damage and dysfunction, and maintain the structural integrity of telomeres. TERF2IP helps to regulate telomere length and prevent the activation of telomere DDR pathways. Note that TERF2IP-TRF2 phosphorylation induces telomere dysfunction and inflammation simultaneously. When TERF2IP is phosphorylated, the TERF2IP-TRF2 complex leaves the Shelterin complex and T-loop, reducing the protective action of the Shelterin complex. Furthermore, TERF2IP-TRF2 is exported into the cytoplasm, where TERF2IP binds to and represses IKK. Repressing this inhibitor of NF-κB induces inflammatory signaling. Therefore, TERF2IP phosphorylation can cause telomere dysfunction and inflammation simultaneously (443). Figure generated using BioRender.com.

TERF2IP associates with TRF2 to bind to telomeres, enhancing TRF2's binding to these structures (438, 439). This complex is essential for inhibiting telomeric recombination, fragility, and shortening (440). Disturbed flow triggers EC activation and senescence by promoting TERF2IP S205 phosphorylation (113, 441, 442). Once phosphorylated at S205, TERF2IP and TRF2 undergo nuclear export. In the cytoplasm, TERF2IP interacts with cytosolic IKK, impairing its ability to inhibit NFκB and leading to EC activation. Concurrently, the removal of TRF2 from the nucleus induces telomere shortening, senescence, and apoptosis (443). This nuclear export of the TERF2IP-TRF2 complex connects inflammatory signaling with telomere shortening, representing a common pathway linking SASP and telomere shortening (443). Taken together, the expression of Shelterin complex and PTMs of TERF2IP play a critical role in regulating EC activation, apoptosis, senescence, and the development of atherosclerotic CVD (Figure 10).

### 1.7. The crucial interplay between nucleus and mitochondria in SASP induction

The interplay between the nucleus and mitochondria is vital for forming the OXPHOS system, comprising protein complexes within the IMM. The nucleus supplies necessary components and regulatory factors for OXPHOS assembly, while mtDNA encodes crucial OXPHOS complex subunits. This coordinated process enables efficient synthesis, assembly, regulation, and maintenance of OXPHOS, facilitating cellular energy production and metabolic adaptation. The nuclear genome controls OXPHOS gene expression in response to cellular conditions, adjusting energy production based on demands and stresses. Nuclear transcription factors respond to nutrients, oxygen levels, and energy status, fine-tuning OXPHOS activity. The nuclear genome also maintain mtDNA integrity and stability, supporting proper mtDNA replication, repair, and protection. Nuclear-encoded subunits, synthesized in the cytoplasm, are imported into mitochondria to combine with mitochondria-encoded subunits. Critical nuclear-encoded factors facilitate import, assembly, and stability of mitochondria-encoded subunits, ensuring proper OXPHOS complexe formation and function (444).

Mice with a mutated mtDNA polymerase *γ* show a premature aging phenotype with increased mtDNA mutation rates (194). Surprisingly, despite the mutations, ROS production and antioxidant enzyme activity remain unchanged, suggesting mtDNA mutations may not significantly contribute to mtROS production or the premature aging phenotype. Other mechanisms, independent of mtDNA damage, may be responsible for the mtROS-mediated premature aging process.

Qian et al. reported that mtROS induce DNA damage in telomeres, activating DDR and peroxisome proliferator-activated receptor (PPAR) pathways (445). Chemoradiation-induced mtROS activates p90RSK, which phosphorylates ERK5 S496, inhibiting the activity of ERK5 and NRF2 transcription. This reduces NRF2 activity (189, 394) and downregulates antioxidant gene expression, including HO1 and TRX1. The decreased NRF2 activity establishes a persistent SASP state with senescence, inflammation, increased mtROS production, and impaired efferocytosis. Low-dose IR or doxorubicin-induced telomeric DNA damage enhances this SASP state.

**Figure 11:**
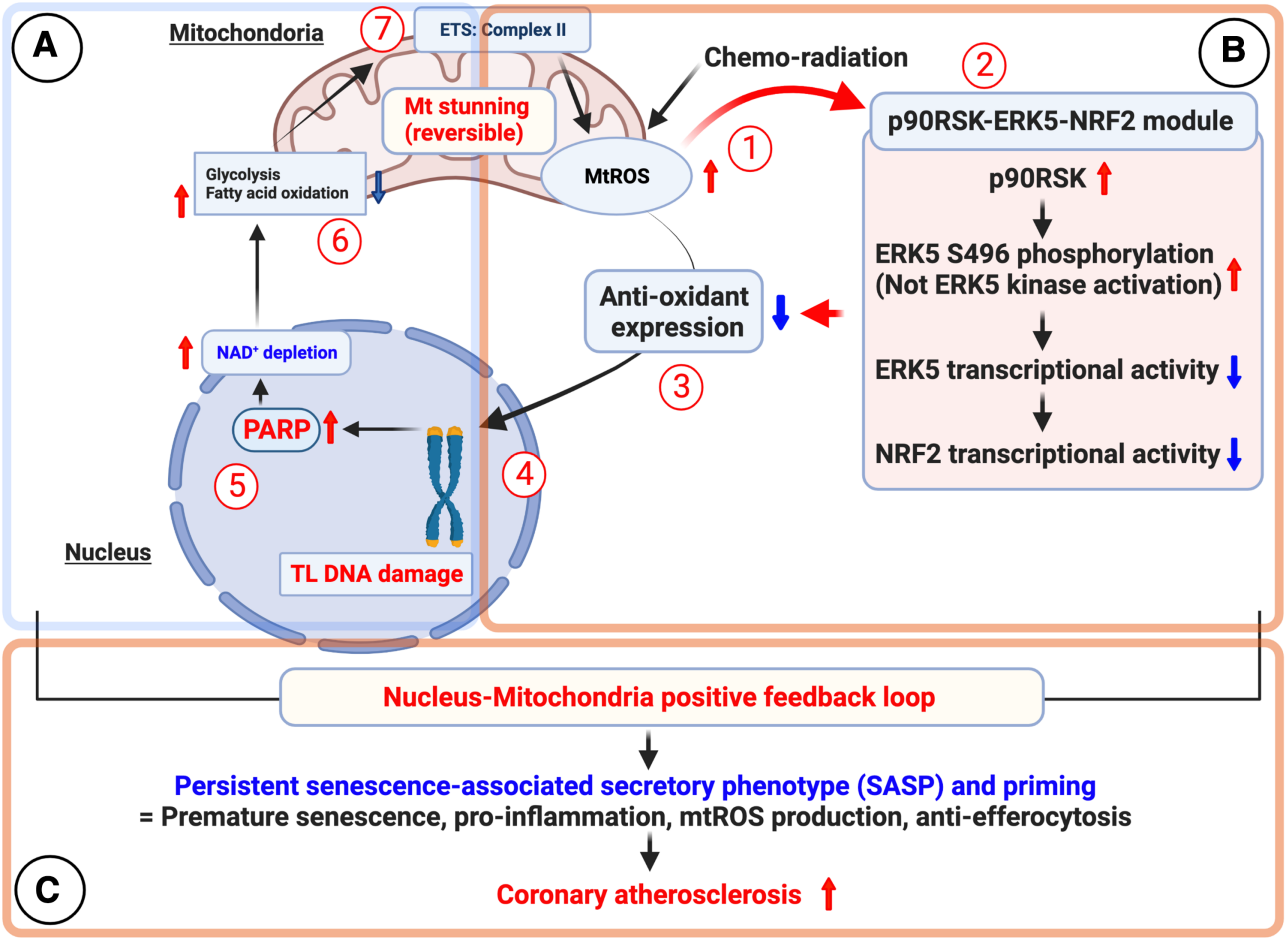
A nucleus-mitochondria positive feedback loop involving the p90RSK-ERK5-NRF2 module and PARP activation [modified from (189)]. (1) Chemo-radiation stimulates mtROS production and phosphorylates p90RSK-ERK5 S496. (2) Subsequently, chemo-radiation inhibits the activity of ERK5 and NRF2 transcription through mtROS-mediated ERK5 S496 phosphorylation. (3) Chemo-radiation decreases antioxidant expression (e.g., HO1 and Trx1), leading to a persistent SASP characterized by senescence, inflammation, mtROS production, and attenuation of efferocytosis. (4) Low dose IR and doxorubicin-induced mtROS production plays a critical role in telomere shortening. (5) Telomere DNA damage activates PARP. (6) Low dose IR and doxorubicin do not cause immediate cell death but lead to “mitochondrial stunning”, a metabolically active state with severe ATP depletion. Mitochondrial stunning induces persistent mtROS production and a late-lasting SASP, detectable long after cancer therapy completion. Schematic created using BioRender.com.

Telomere DNA damage activates PARPs, depleting NAD^+^ and causing mitochondrial dysfunction (338). Chemoradiation treatment increases succinate levels and mtROS production via complex II. “Mitochondrial stunning” refers to a reversible mitochondrial dysfunction with severe ATP depletion without immediate cell death (189). This phenomenon activates the p90RSK-ERK5-NRF2 signaling pathway, establishing a positive feedback loop between mitochondrial dysfunction and nuclear DDR. This loop sustains mtROS production, contributing to premature aging through telomere erosion (189) (Figure 11).

Transient inhibition of PARP during radiation exposure has been shown to reduce IR-induced coronary atherosclerosis, macrophage infiltration, and cardiac dysfunction (189, 446). However, long-term PARP inhibition may increase chromosomal instability and tumorigenesis risk, making it unsuitable for prolong clinical use as an anti-atherosclerosis drug (447). The data suggest that transient PARP inhibition specifically during RT effectively prevent cardiovascular damage without the potential tumorigenesis risk associated with prolonged PARP inhibition. This preclinical evidence supports using transient PARP inhibition as a therapeutic strategy to mitigate radiation-induced cardiovascular damage (189). These findings emohasize the crucial interplay between the nucleus and mitochondria in inducing SASP. Without this positive feedback loop, sustained SASP induction cannot occur, leading to premature aging-related CVD, especially following various cancer treatments.

## 2. Discussions

The rising prevalence of CVD among older patients and cancer survivors has become a significant concern due to the expanding population of these individuals. Managing CVD in these groups pose challenges, given the complex molecular and intracellular events associated with cellular senescence (113). Senescence and premature senescence processes play both protective and detrimental roles in DNA damage-associated tumorigenesis and CVD. These events involve persistent DNA damage, chronic activation of DDR pathways, telomere dysfunction, mitochondrial dysfunction, metabolic and epigenetic alterations, mtROS production, SASP, and SAS (132, 335, 374–385). Therefore, it is crucial to carefully weigh the benefits and potential risks.

Chemotherapy (40) and RT, commonly cancer treatments, induce DNA damage and oxidative stress, leading to SIPS in various cardiovascular system cells (cardiomyocytes, ECs, myeloid cells). Both telomeric and non-telomeric DNA damage contribute to cellular senescence, but dysfunction, not telomere length, better reflects SIPS due to less efficient telomeric DDR repair compared to genomic DDR. Persistent telomeric DNA damage leads to the formation of TAFs, marking cellular senescence. Senescent cells (RS or SIPS) secrete pro-inflammatory factors known as SASP. Mitochondrial dysfunction plays a crucial role in SIPS and SASP induction through factors such as NAD^+^ depletion, increased mtROS production, impaired mitochondrial fission/fusion, and compromised mitophagy. Telomeric DDR activation and TAF formation, along with epigenetic changes, can create a nucleus-mitochondrial positive feedback loop, facilitated by PARP-mediated NAD^+^ depletion. Cancer therapies can activate this loop through a p90RSK-ERK5 module, resulting in chronic inflammation and tissue remodeling. Ultimately, these processes can contribute to CVD development after cancer treatments.

Our recent research has provided significant insights into the regulation of SASP and SAS in myeloid cells (392, 448). We found that p90RSK-mediated ERK5 S496 phosphorylation governs factors contributing to SASP (189), including mtROS induction, telomere shortening-triggered DNA damage, activation of p16INK4, p21Cip1/Waf1, p53, inflammation, and impaired efferocytosis in HIV patients on antiretroviral therapy (448). Cancer treatments can also induce SASP, linked to cancer cell proliferation treatment resistance. Our study demonstrated the important of p90RSK activation in immunosenescence (370) and atherosclerosis inhibition (394), indicating the potential significance of p90RSK-mediated ERK5 S496 phosphorylation in SASP and SAS regulation, and its implications for CVD and cancer treatment resistance. Further investigations are needed to fully understand the mechanisms of SASP modulation by p90RSK-mediated ERK5 S496 phosphorylation (392, 448). Additionally, research should explore the role of the p90RSK-ERK5-NRF2 axis in ECs. The interplay between SASP induced by cancer treatment and p90RSK-mediated ERK5 S496 phosphorylation may accelerate coronary atherosclerotic plaque formation.

Oncogene-induced senescence (OIS) occurs when non-tumor cells activate oncogenes, leading to stable cell cycle arrest (357). Recent research by Leon et al. discovered that OIS and overexpression of the oncogene HRASG12V in IMR90 cells increase active histone H3K79 di- and tri-methylation (H3K79me2/3) specifically at the IL1A gene locus (449). The histone methyltransferase disruptor of telomeric silencing 1-like (DOT1l) regulates this change, crucial for IL1A expression during OIS, but not directly involved in SASP. While epigenetic changes may contribute to irreversible SASP state in OIS cells (450, 451), their specific role in regulating SAS remains unknown. Further investigations are needed to understand epigenetic factors and their contribution to the SAS phenotype. Additionally, future research should explore other stressors inducing both SASP and SAS, broadening our knowledge of these cellular processes.

Senescence is linked to impaired angiogenesis (96), a critical process for forming new blood vessels. Inhibiting telomerase in ECs reduces angiogenesis in tumor and hind limb ischemia models. Targeting senescent cells offers a promising therapeutic approach for managing conditions with impaired angiogenesis (452, 453). Cancer treatment induces senescence in ECs (454), but the role of EC’s SASP in the delayed onset of CVD after cancer treatment remains unclear. Further research is needed to explore this aspect and understand the interplay between EC senescence, the SAS phenotype, and CVD in cancer survivors (113). These investigations will provide valuable insights into the cardiovascular consequences of cancer treatment and guide strategies for preventing and managing CVD in this population.

## Data Availability

The original contributions presented in the study are included in the article/Supplementary Material, further inquiries can be directed to the corresponding author/s.
